# Effects of cadmium on the synthesis of active ingredients in *Salvia miltiorrhiza*

**DOI:** 10.1515/biol-2022-0603

**Published:** 2023-05-24

**Authors:** Haihui Fu, Jun Yuan, Rongpeng Liu, Xiaoyun Wang

**Affiliations:** Key Laboratory of Crop Physiology, Ecology and Genetic Breeding, Ministry of Education, Jiangxi Agricultural University, Nanchang, China; School of Nursing, Jiangxi University of Chinese Medicine, Nanchang, China; School of Pharmacy, Jiangxi University of Chinese Medicine, Nanchang, China; Research Center for Traditional Chinese Medicine Resources and Ethnic Minority Medicine, Jiangxi University of Chinese Medicine, Nanchang, China

**Keywords:** *S. miltiorrhiza*, Cd stress, Cd enrichment, physiological characteristics, metabolic profiles

## Abstract

Cadmium (Cd) could pose threats to human health by affecting *Salvia miltiorrhiza* (SM) safety. Cd enrichment trait and its effects on the active ingredient synthesis in SM remain unknown. Here we investigated the Cd concentration using ICP-MS-based method, physiologies (contents of malondialdehyde and proline, and activities of superoxide dismutase, peroxidase [POD], and catalase [CAT]), and LC-MS/MS-based metabolites of SM under 25, 50, and 100 mg kg^−1^ Cd stress. The results revealed that Cd concentrations, as it rose in soil, increased in roots and leaves of SM with transfer factors and bioconcentration factors below 1 in Cd-treated groups; POD and CAT activities and proline content increased and then declined. Amino acids and organic acids (especially d-glutamine [d-Gln], l-aspartic acid [l-Asp], l-phenylalanine [l-Phe], l-tyrosine [l-Tyr], geranylgeranyl-PP [GGPP], and rosmarinic acid [RA]) contributed more in discriminating SM roots of different groups. GGPP was negatively related to l-Tyr and l-Phe, and RA was positively related to d-Gln and l-Asp in SM. These results revealed that SM belonged to a non-Cd-hyperaccumulator with most Cd accumulated in roots, Cd could enhance phenolic acid synthesis via regulating amino acid metabolism and might inhibit tanshinone synthesis by declining the GGPP content, and proline, POD, and CAT played vital roles in resisting Cd stress. These provided new ideas and theoretical basis for further study on medical plants’ response to heavy metals.

## Graphical abstract

**Figure:**
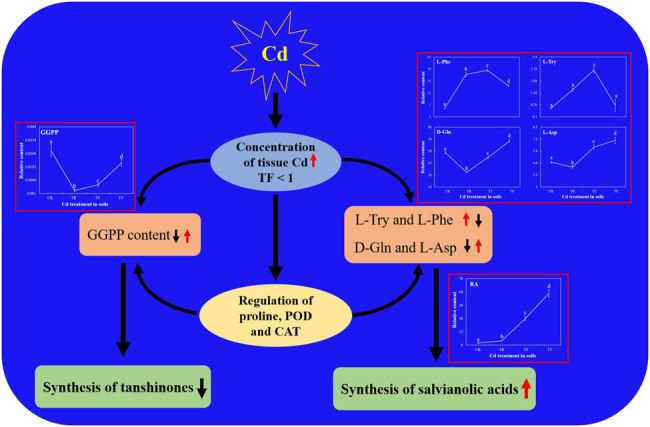

## 1 Introduction

With the booming of the economy and society, serious cadmium (Cd) pollution emerged in soils, which is caused by the unreasonable production in the long-term mining and smelting, and unreasonable farming in traditional agriculture growth [1,2]. Characterized by strong bioaccumulation, high bioavailability, and strong biotoxicity, Cd migrates easily from soil to plants, which can lead to the decline of plant yield and quality [3]. Eventually, Cd enters the human body via food chains, and seriously threatens people’s health [4]. Hence, much attention has been paid to soil Cd contamination [5,6].

The plant Cd concentration would increase under Cd stress. The absorbed Cd can break the balances of production and elimination of reactive oxygen species (ROS), change the physiological state (e.g., contents of malondialdehyde [MDA], proline, and soluble protein), and affect diverse metabolism systems [7,8,9]. And plants would regulate enzyme and non-enzyme-based antioxidant system, and metabolisms of sugars, amino acids, and organic acids to ameliorate Cd stress [7,10,11]. For instance, the MDA and proline concentrations, activities of SOD, POD, and CAT, and contents of sugars, amino acids, and organic acids changed significantly in maize (*Zea mays*) and wheat (*Triticum aestivum*) with Cd treatment compared with those of the control (without Cd) [12,13,14,15].

*Salvia miltiorrhiza* (SM) is a perennial plant and widely distributed in north and south China [16]. The dry roots and rhizomes (called Danshen in China) of SM contain tanshinone, salvianolic acid, rosemary, and other medicinal ingredients [10]. Nowadays, SM has been widely used to treat cerebrovascular and cardiovascular diseases, hyperlipidemia, and acute ischemic stroke in China and other Asian countries [10,17,18]. With the increasing need for Danshen, the planting area of SM has expanded year by year, which increases their risk of being contaminated. SM from some production areas contained Cd concentration above the limits set by the Chinese Pharmacopoeia Commission [3]. Much attention should be paid on the research of SM response to Cd.

The previous study has found that Cd could debase the quality of SM by inhibiting the accumulation of active ingredients [19]. However, little is known as relates to Cd enrichment trait and the effect of Cd pollution on the synthesis of active ingredients in SM. In this study, SM was used as the samples and a pot-culture experiment was conducted. The queries to be addressed are as follows: (1) What is the Cd enrichment laws of SM? (2) How does Cd affect the synthesis of active ingredients in SM based on metabolisms and physiologies? The study would assist with understanding how SM responds to Cd stress physiologically and metabolically, and provide new idea for the research on mechanism of SM in response to Cd.

## 2 Materials and methods

### 2.1 Soil preparation

The soil used in the pot experiment was collected from the topsoil (0–20 cm) in Shennong Garden of the Jiangxi University of Chinese Medicine in Jiangxi, China (E 115°44′24″, N 28°41′24″). The soil was air-dried, sieved to remove gravels through a 4 mm mesh sieve, and then mixed thoroughly. The basic physicochemical properties, including total nitrogen [N], available N, total phosphorus [P], available P, total potassium [K], available K, total Cd concentration, organic matter content, and pH value, were described in previous study [20]. The total Cd concentration was 0.95 mg kg^−1^, which is less than the critical value (1.0 mg kg^−1^) of the soil to ensure normal plant growth in agriculture and forestry production [21].

The appropriate contents of cadmium chloride hemi (pentahydrate) (CdCl_2_·2.5H_2_O, Sinopharm Chemical Reagent Co., Ltd, China) were thoroughly blended with soil to obtain the final concentrations of 25, 50, and 100 mg Cd per kilogram of soil, respectively. The selected Cd concentrations were consistent with that of previous studies of Wang et al. [22] and Zhang et al. [19]. Then, the soils with and without Cd treatment were transplanted into plastic pots (16 cm in diameter × 17 cm in height) and watered every 5 days with pure water. Before use, they were equilibrated for 30 days.

### 2.2 Plant material and Cd treatment

The source and treatment ways of SM seedlings were as per the study of Yuan et al. [13,20]. SM seedlings were purchased from SM planting areas (Shangdong, China). The formal identification of the samples was carried out by Assoc Prof. Xiaoyun Wang. Cultivated in Shennong garden for 30 days, the seedlings were transferred into appropriate pots containing 2.5 kg soil. Each pot contained two seedlings. A pot was taken as one replicate, and three replicates were performed for each treatment. In the experiment, seedlings without Cd treatment were used as the control group, and seedlings with 25, 50, 100 mg kg^−1^ Cd treatment were named TR, TS, and TT, respectively. The selected Cd concentrations were consistent with that of previous studies of Wang et al. [22] and Zhang et al. [19]. The experiment was conducted under non-greenhouse conditions. Each seedling presented uniform growth. After 15 days, plant samples were harvested for further study.

In order to perform LC-MS and physiological analyses, root samples from each pot were selected to make a composite root sample, simultaneously frozen in dry ice and stored at −80°C. Leaves and remaining roots from each pot were collected to make a composite leaf sample and root sample, respectively, to perform Cd concentration analysis. Then, both samples were dried in the oven at 60°C for 4 days. Voucher specimens (NO. DS-001) were deposited in a public herbarium in Research Center for Traditional Chinese Medicine Resources and Ethnic Minority Medicine of Jiangxi University of Chinese Medicine [20].

### 2.3 Determination of Cd concentration

The oven-dried samples were ground to fine powder, passed through a 2 mm sieve, and then digested with a mixture of nitric acid (HNO_3_) and hydrogen peroxide (H_2_O_2_) (3/1, v/v) in Teflon tanks using an electric heating board at 160℃ thoroughly. The Cd concentration was determined by inductively coupled plasma mass spectrometry (ICP-MS; Thermo Scientific, USA).

### 2.4 Physiological analysis

The ninhydrin colorimetry was used to determine the proline content [23,24]. A 0.05 g fresh root sample was pooled in the centrifugal tube with 5 mL of 3% sulfosalicylic acid, extracted in the boiling water bath for 10 min, and shaken frequently. The extraction was filtered into a volumetric flask. Then, the volume was constant to 25 mL with distilled water. A total of 2 mL of extraction solution mixed with 2 mL of glacial acetic acid and 2 mL of acidic ninhydrin was added in a centrifuge tube. The mixture was treated with the boiling water bath for 30 min. After cooling down to room temperature, 4 mL of toluene was added to the mixture and shaken thoroughly. When they were stratified during standing, the absorbance of the upper solution was measured at 520 nm with an ultraviolet spectrophotometer (UV-8000, Metash, China).

The content of MDA and activities of peroxidase (POD), superoxide dismutase (SOD), and catalase (CAT) were detected by assay kits (Suzhou Keming, China) [13]. A total of 0.1 g fresh samples and 1 mL solutions (1/10, v/v) were grounded in a water bath and centrifuged at 8,000 × *g* and 4℃ for 10 min. According to the manufacturer’s instructions, the supernatant absorbance was measured at 532 and 600 nm to assess MDA content, and at 470, 240, and 560 nm to evaluate the activities of POD, CAT, and SOD, respectively.

### 2.5 Metabolite analysis

The method of metabolite extraction was carried out as reported by Wang et al. [25] and Yuan et al. [13]. A total of 25 mg fresh samples was placed in a microcentrifuge tube with 500 μL of extract solution (methanol:water = 3:1 (v/v), with the isotopically labeled internal standard [IS] mixture), homogenized at 35 Hz for 4 min and sonicated for 5 min with an ice-water bath. The homogenization and sonication cycle were conducted three times. The samples were incubated for 1 h at −40℃ and centrifuged at 12,000 rpm and 4℃ for 15 min. Subsequently, the resulting supernatant was transplanted into a fresh glass vial for further analysis. Besides, the quality control (QC) sample was formed by mixing an equal aliquot of the supernatants from all samples [26].

Combining a UHPLC system (Vanquish; Thermo Fisher Scientific, USA) and Q Exactive HFX mass spectrometer (Orbitrap MS; Thermo, USA), LC-MS/MS analyses were performed to analyze sample metabolites using a Waters ACQUITY UPLC HSS T3 (2.1 mm × 100 mm, 1.8 μm; Waters, USA). Mobile phase A was water containing 5 mmol L^−1^ ammonium acetate and 5 mmol L^−1^ ammonia hydroxide, while mobile phase B was acetonitrile. The analysis was carried with elution gradient as follows: 0–0.7 min, 1.0% B; 0.7–9.5 min, 1.0–99% B; 9.5–11.8 min, 99% B; 11.8–12.0 min, 99–1% B; 12–14.8 min, 1.0% B. The column temperature was 35℃. The auto-sampler temperature was 4℃, the injection volume was 3 μL, and the flow rate was 0.5 mL min^−1^. The QE HFX mass spectrometer was conducted to acquire MS/MS spectra based on information-dependent acquisition mode in controlling the acquisition software (Xcalibur; Thermo, USA). In the mode, the acquisition software continuously evaluated the full scan MS spectrum. The ESI source conditions were conducted as follows: the flow rates of sheath gas and Aux gas were 30 Arb and 10 Arb, respectively, the capillary temperature was 350℃, the full MS resolution was 60,000, the MS/MS resolution was 7,500, the collision energy was 10/30/60 in NCE mod, and the spray voltage was 4.0 kV (positive) or −3.8 kV (negative).

After the original data were converted into mzXML format using the software ProteoWizard (https://proteowizard.sourceforge.io/), the R package XCMS (version 3.2) was used for the peak recognition, extraction, alignment, and integration. The preprocesses of the original data included the following: (1) Data filtering: The filtering standard was to remove the data with no definite substance name or no spectrum comparison similarity. (2) Missing values processing: Substances of more than 50% missing in comparisons were filtered directly, and substances of less than 50% missing were performed the imputation of missing values using the k-nearest neighbor algorithm. (3) Normalization: The IS or total ion current of each sample was used for the normalization. A total of 1,111 and 305 peaks of the original data were retained for positive and negative ion modes, respectively. The excel sheets, including the name of peak and sample, and the standard data of normalized peak area were obtained for further data analysis.

### 2.6 Statistical analysis

The changes in the Cd enrichment, and physiological and metabolic traits were presented in the study coupled with the results of the previous study [13,20]. First, the transfer factor (TF) and bioconcentration factor (BCF) of Cd were calculated as follows [27,28,29]:

TF = Cd concentration of aboveground parts (mg kg^−1^)/Cd concentration of roots (mg kg^−1^),

BCF = Cd concentration in the tissues/Cd concentration in soils.

Second, the multivariate ordination principal component analysis (PCA) was conducted to reveal the overall distribution of the samples. The supervised partial least squares discrimination analysis (PLS-DA) was utilized to assess the differences in roots between the control and Cd treatment groups. The PLS-DA model was validated by permutation tests (200) or ANOVA of the cross-validated residuals (CV-ANOVA). A Q2 value of above 0.5 and an R2 value of above 0.7 for the permutation test or *p* value of below 0.05 for the CV-ANOVA denoted the highly significant model [30]. The variable importance in projection (VIP) was crucial for explaining the data of the PLS-DA model. Metabolites with a *p* value of below 0.05 and a VIP of above 1.0 were filtered out as differential metabolites, playing larger roles in distinguishing roots between the control and Cd addition groups [20].

Third, the pathway analysis of all the metabolites was conducted by the software MetaboAnalyst 4.0 (http://www. metaboanalyst.ca/faces/ModuleView.xhtml). Based on the pathway impact value of above 0.1, the potential metabolic target pathways were obtained [20,31]. The Venn diagram was obtained using the software Venny 2.1 (http://bioinfogp.cnb.csic.es/tools/venny/) to present the overlap of all the named metabolites in the control and Cd treatment groups. Subsequently, t-test and correlation analyses were conducted to evaluate the variability of identical metabolites in samples, and relationships between Cd concentration in tissues (leaves and roots) and Cd concentration in soils and relationships among Cd concentration, contents of Pro and MDA, activities of SOD, CAT, and POD, and relative contents of d-glutamine (d-Gln), l-aspartic acid (l-Asp), l-tyrosine (l-Tyr), l-phenylalanine (l-Phe), geranylgeranyl-PP (GGPP), and rosmarinic acid (RA) in roots with SPSS 18.0 (SPSS Inc., USA), respectively. The PCA and PLS-DA models were performed by SIMCA-P version 14.1 (Umetrics, Sweden). All data were log10-transformed before analysis.

## 3 Results

### 3.1 Cd concentration in leaves and roots

**Figure 1:**
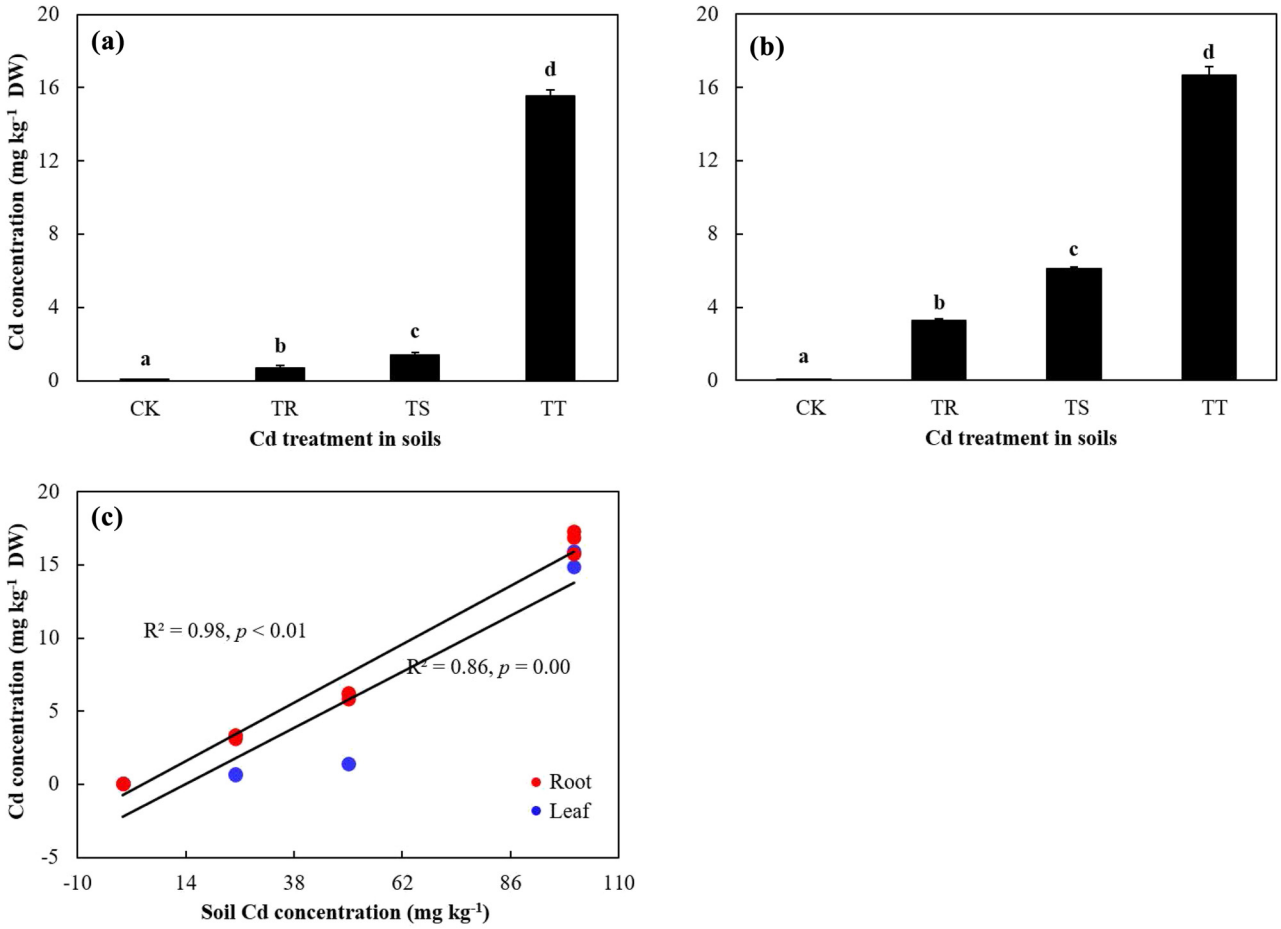
Enrichment characteristics of Cd in leaves and roots of *S. miltiorrhiza* with different levels of soil Cd stress. (a) and (b) Cd concentration in SM leaves and roots with different levels of soil Cd stress, respectively, and the data are presented as the mean value ± SE (*n* = 3); (c) relationships of Cd concentration in leaves and roots and soil Cd concentration, respectively; different letters indicate significant differences among different treatments.

With the increase in soil Cd concentration, Cd concentrations in leaves and roots increased significantly (*p* < 0.05) (Figure 1a–c). Under 100 mg kg^−1^ Cd stress, Cd concentrations both in leaves and roots reached the maximum (15.53 mg kg^−1^ for leaves and 16.66 mg kg^−1^ for roots), which were 99.68 and 99.70% higher than that in the control group, respectively (Figure 1a and b).

Besides, with the increase in soil Cd concentration, the TF and BCF of SM changed significantly (*p* < 0.05). The max-TF was presented in roots of the control group (Table 1). As soil Cd concentration rose, the seedling BCF value decreased first, then increased, and finally declined significantly (*p* < 0.05), while the max-BCF value was also in the control group (Table 1). With the increase in soil Cd concentration, the BCFs in roots increased. The BCFs of tissue samples with 100 mg kg^−1^ Cd treatment were higher than those in other groups (*p* < 0.05) (Table 1). All TFs and BCFs were below 1 except the TF value in roots of the control group (Table 1).

**Table 1:** TF and BCF of SM under different levels of Cd stress

Cd treatment	TF	BCF of roots	BCF of leaves	BCF of the seedlings
CK	1.00 ± 0.02^a^	0.05 ± 0.00^a^	0.05 ± 0.00^a^	0.95 ± 0.00^a^
TR	0.21 ± 0.01^b^	0.13 ± 0.00^b^	0.03 ± 0.00^b^	0.43 ± 0.01^b^
TS	0.23 ± 0.00^b^	0.12 ± 0.00^b^	0.03 ± 0.00^b^	0.65 ± 0.01^c^
TT	0.93 ± 0.04^a^	0.17 ± 0.00^c^	0.16 ± 0.00^c^	0.52 ± 0.01^d^

CK, TR, TS, and TT stand for roots in the control, 25 mg kg^−1^ Cd, 50 mg kg^−1^ Cd, and 100 mg kg^−1^ Cd group, respectively (the same below). The data were presented as the mean value ± SE (*n* = 3). The data with different little letters in same column show significant difference (*p* < 0.05).

### 3.2 Physiological characteristics in roots

**Figure 2:**
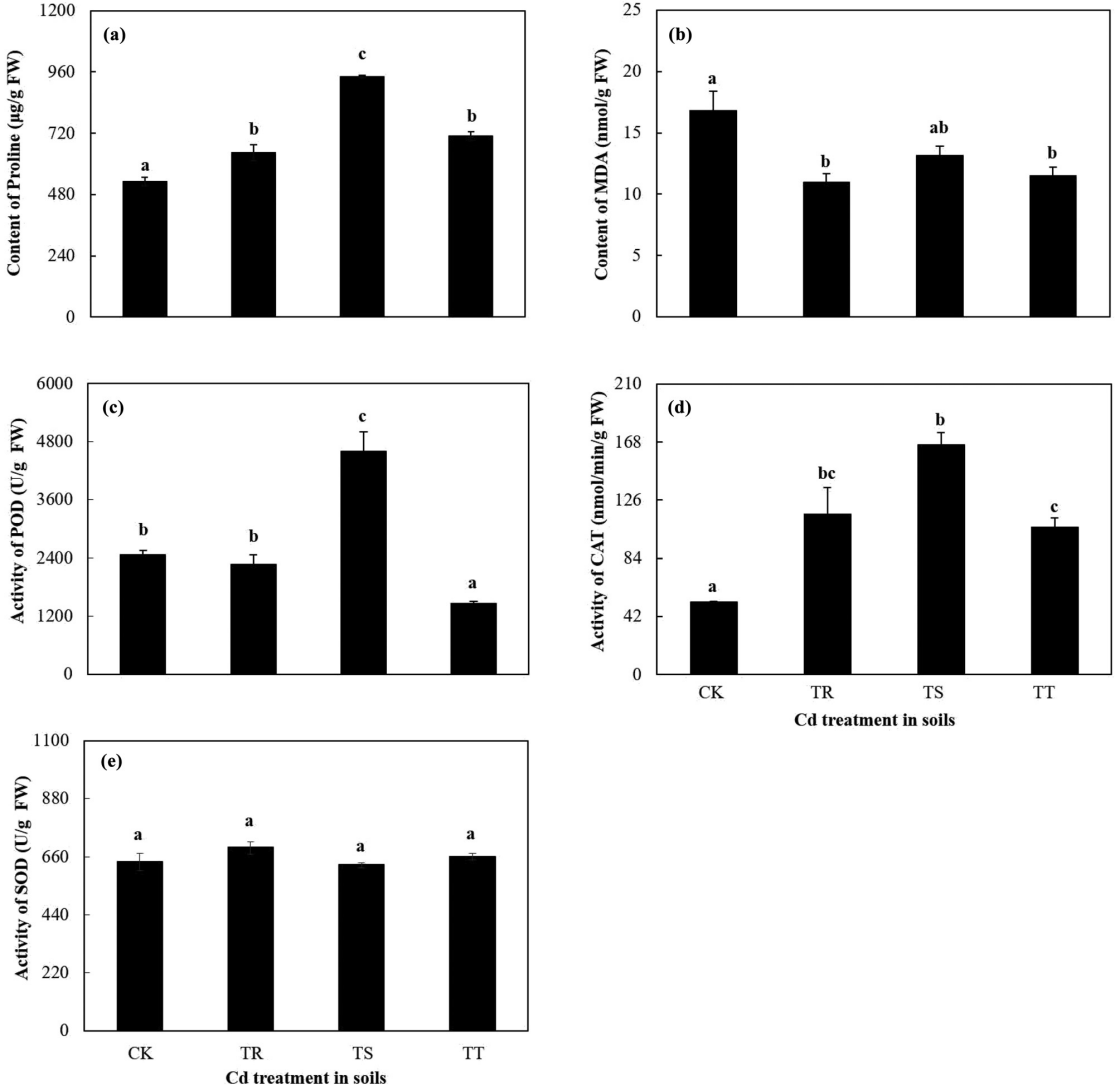
Physiological characteristics of SM roots with different levels of soil Cd stress. (a) and (b) Effect of Cd on the content of proline (a) and MDA (b) in SM roots, respectively. (c)–(e) Effect of Cd on the activities of POD, CAT, and SOD in SM roots, respectively. The data were presented as the mean value ± SE (*n* = 3); different letters indicate significant differences among different treatments.

The proline content in roots went up significantly at first and then went down significantly with an increase in soil Cd concentration (*p* < 0.05). The proline content in 50 mg kg^−1^ Cd added group was higher than other groups (*p* < 0.05) (Figure 2a). But as soil Cd concentration increased, the MDA content declined (Figure 2b). The activities of POD and CAT in roots increased and then decreased significantly with the increase in soil Cd concentration (*p* < 0.05) (Figure 2c and d). The maximal activities of POD and CAT both appeared in roots of 50 mg kg^−1^ Cd added group (Figure 2c and d). However, the SOD activity of roots revealed no significant differences in different levels of Cd-contaminated groups (Figure 2e).

### 3.3 Metabolomic changes in roots

**Figure A1:**
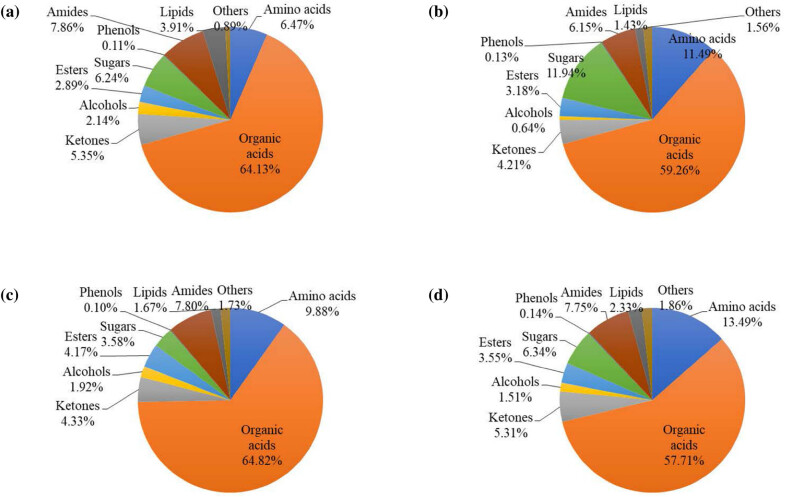
Classification characteristics of metabolites in SM roots with different levels of soil Cd stress. (a–d) Classification characteristics of metabolites in SM roots of the control, 25, 50, and 100 mg kg^−1^ Cd group, respectively.

A total of 305 metabolites were identified, as shown in the previous study (Table S1). Most of them were C-containing and N-containing metabolites (e.g., amino acids, organic acids, fatty acids, etc.). The relative contents of these metabolites of the control group differed from those in Cd-treated groups (Figures 4 and 5; Figure A1; Table S1). Besides, a metabolic map was obtained according to pathway analysis results, involving all the identified metabolites (Figure 5). The target pathways (with the PI value of above 0.1) are presented in Table S3, coupled with amino acid metabolism and some secondary metabolism as the central metabolic pathways (Table S2). Some of them were involved in the synthesis of active ingredients (Figure 5).

**Figure 3:**
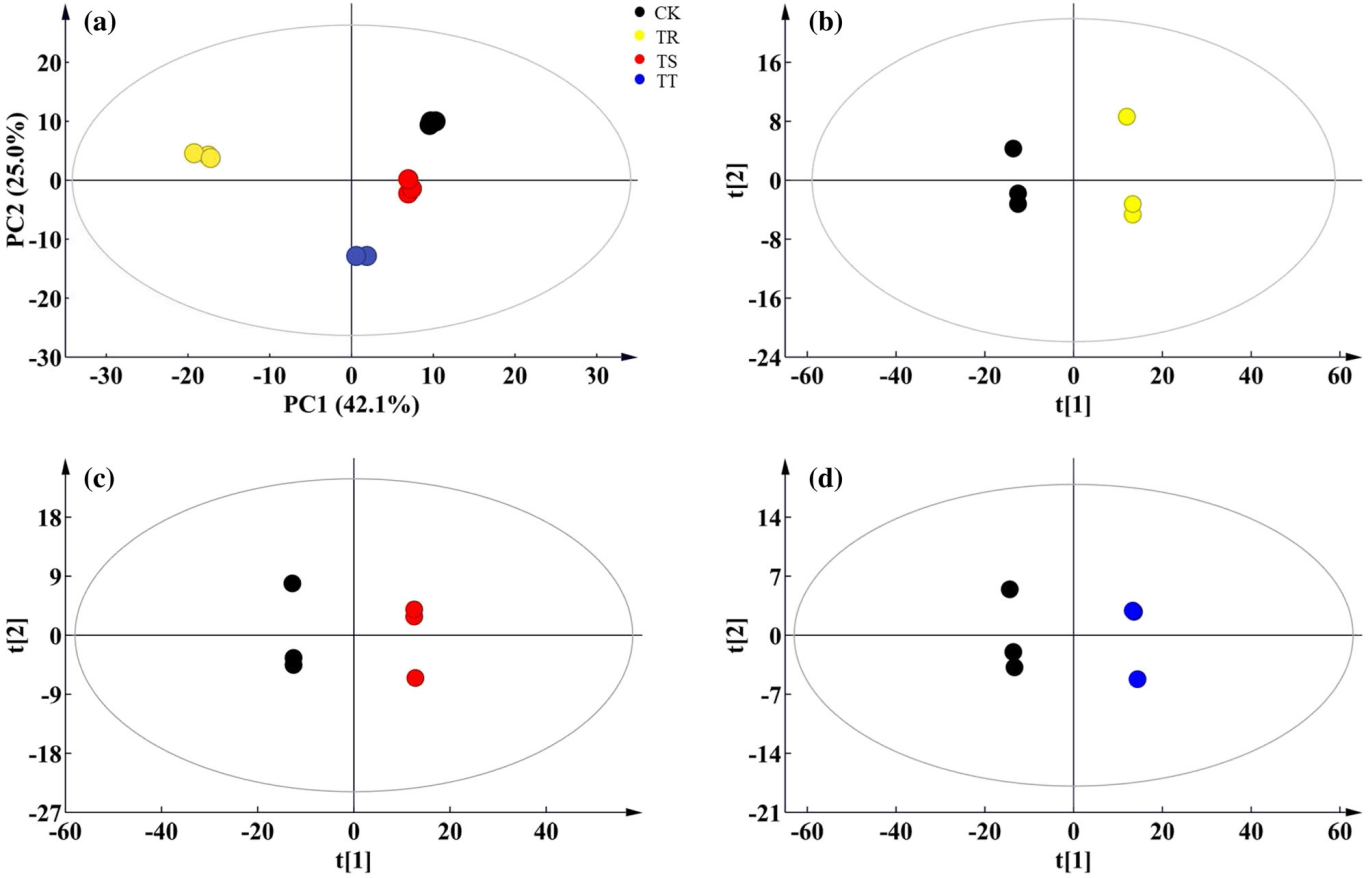
Changes in metabolites in SM roots under different levels of Cd stress. (a) The score plots of PCA for metabolomic data from SM roots under different levels of Cd stress PC1, the first principal component; PC2, the second principal component. The ellipse indicates the Hotelling’s T2 (95%); (b–d) score plots of PLS-DA for metabolomic data from SM roots of the control and the 25 mg kg^−1^ (b), 50 mg kg^−1^ (c), and 100 mg kg^−1^ (d) Cd group, respectively.

**Figure 4:**
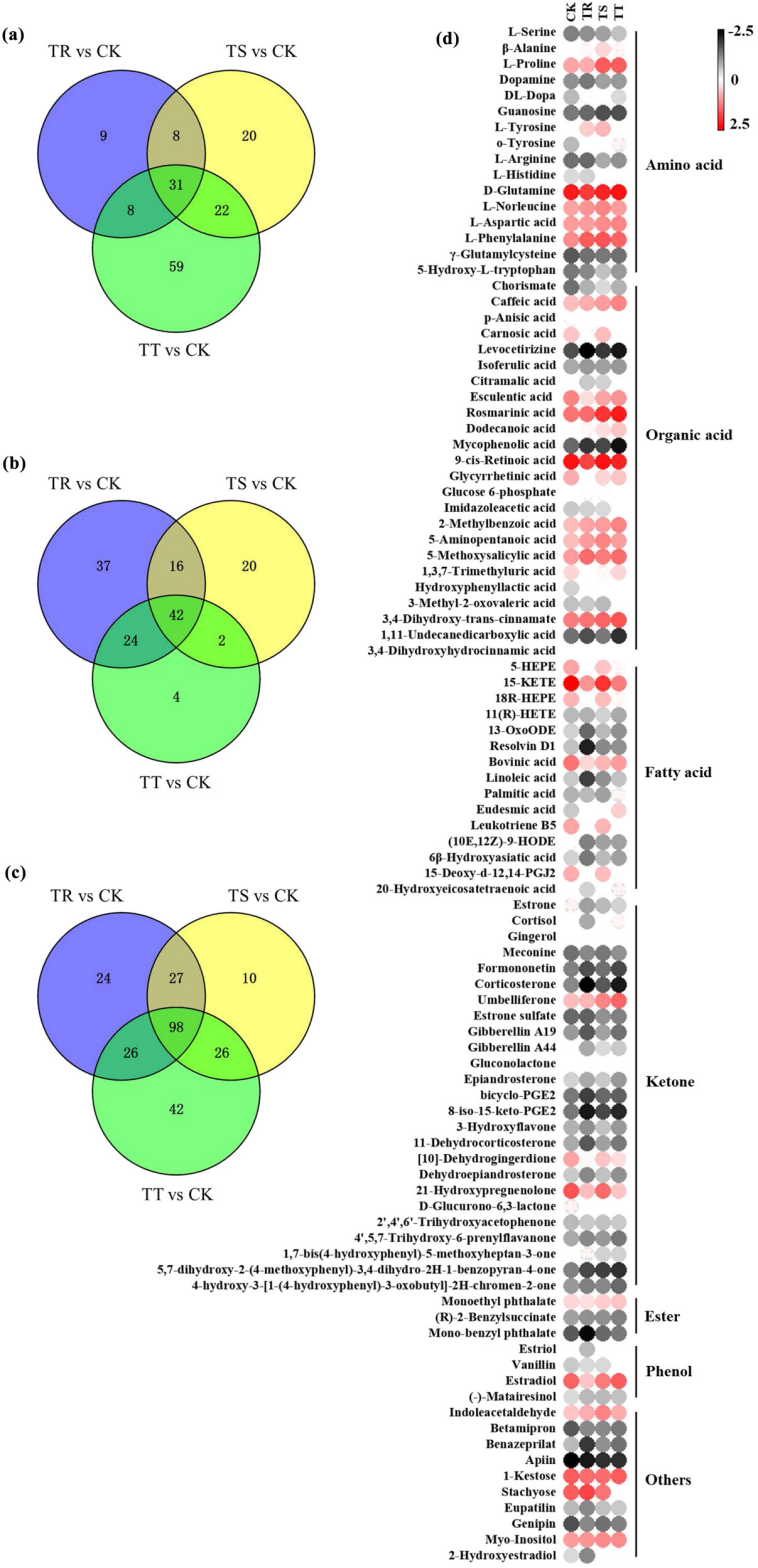
Variation in the differential metabolites in SM roots under different levels of Cd stress. (a–c) Overlap of the up-differential metabolites, down-differential metabolites, and all the differential metabolites of SM roots in response to different Cd stress, respectively. (d) Heatmap analysis of the same differential metabolites in SM roots in response to different Cd stress.

**Figure 5:**
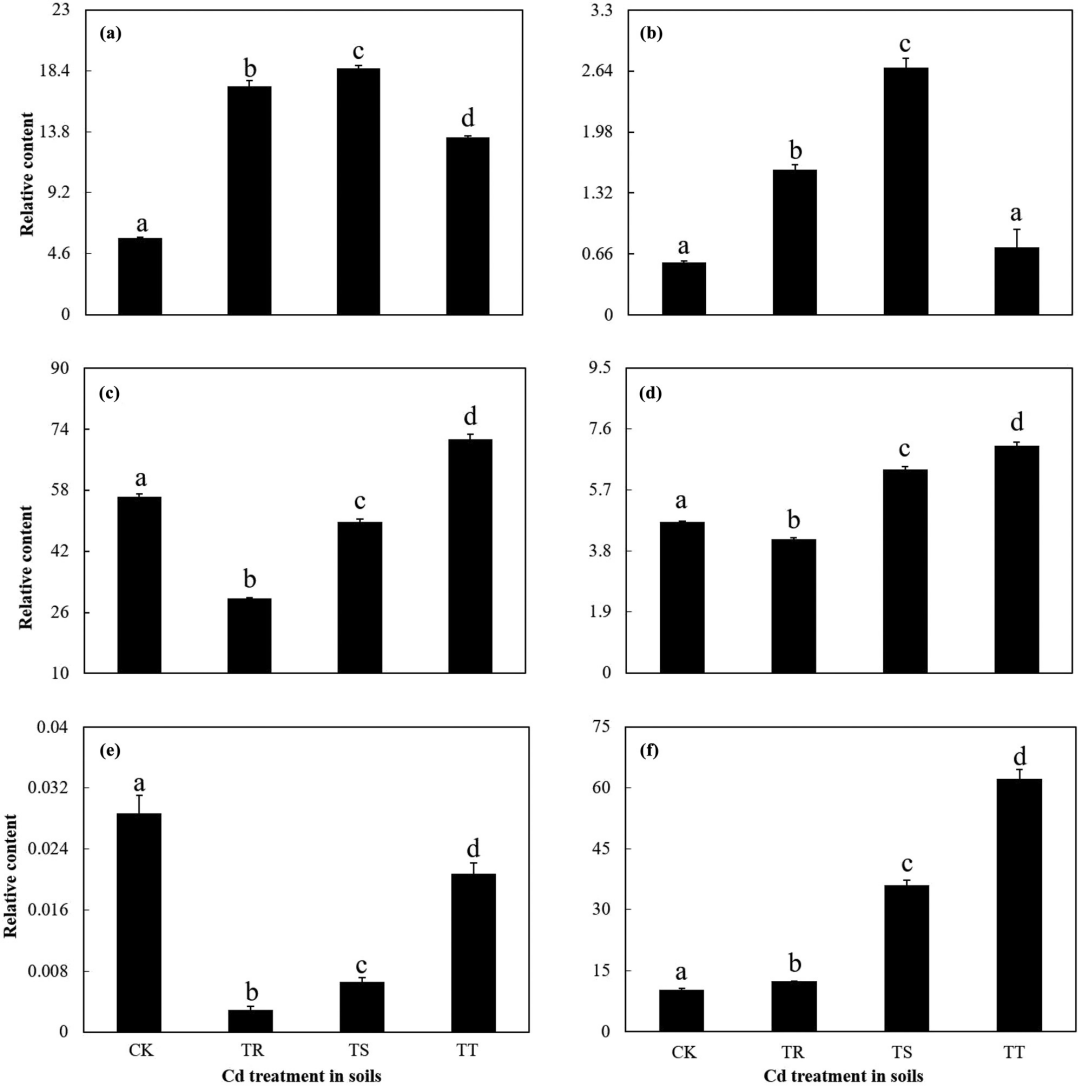
Relative contents of l-Phe (a), l-Tyr (b), d-Gln (c), l-Asp (d), GGPP (e), and RA (f) of SM roots under different levels of Cd stress. The data are presented as the mean value ± SE (*n* = 3); different letters indicate significant differences among different treatments.

### 3.4 Differential metabolites in roots

**Figure A2:**
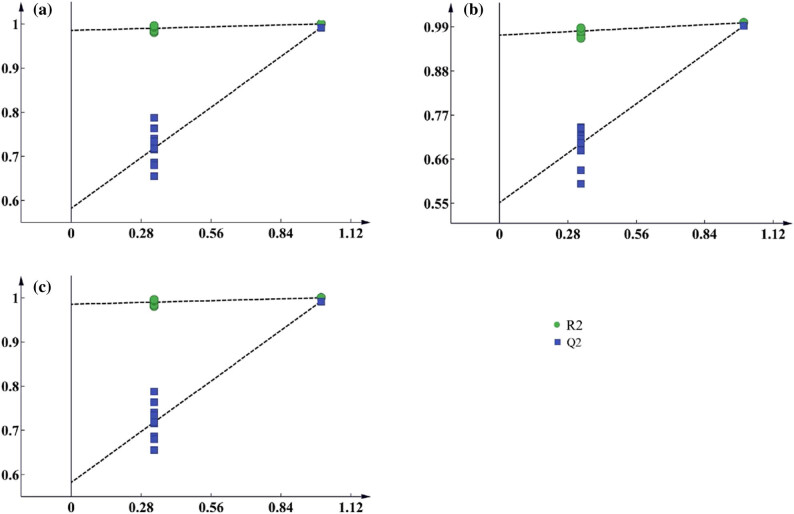
Permutation test results of PLS-DA models for metabolomic data from SM roots. (a–c) The permutation test results of PLS-DA models for roots of the control and 25 mg kg^−1^ Cd group (R2Y[1] = 0.99, Q2[1] = 0.58, CV-ANOVA *p* = 0.03), of the control and 50 mg kg^−1^ Cd group (R2Y[1] = 0.97, Q2[1] = 0.54, CV-ANOVA *p* = 0.03) and of the control and 100 mg kg^−1^ Cd group (R2Y[1] = 0.93, Q2[1] = 0.47, CV-ANOVA *p* = 0.01), respectively.

According to the result of PCA analysis and PLS-DA models, root samples in the control and Cd treatment groups were clearly distinguished in the pot experiment (Figure 3). The results of the significant PLS-DA model (with VIP of above 1) and the *t*-test (with a *p* value below 0.05) (Figure 3b–d and Figure A2) facilitated the identification of differential metabolites, with 175 differential metabolites for the discrimination of the control group and 25 mg kg^−1^ Cd-added group, 161 for the discrimination of the control group and 50 mg kg^−1^ Cd-added group, and 192 for the discrimination of the control group and 100 mg kg^−1^ Cd-added group (Figure 4; Tables S1 and S2).

**Figure 6:**
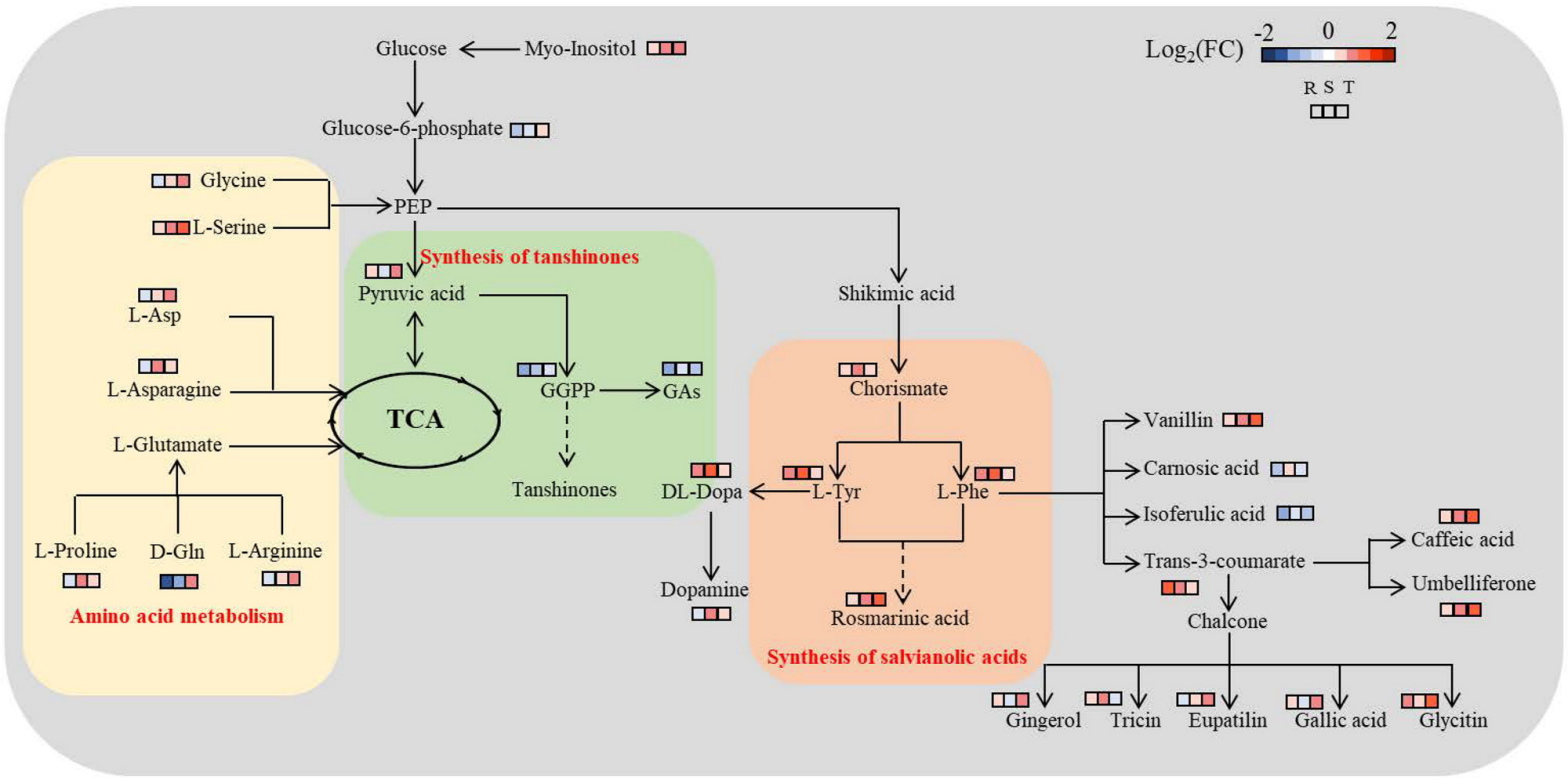
Schematic diagram of the metabolic pathways under different levels of Cd stress. l-Tyr, l-Phe, GGPP, and GAs stand for l-Tyrosine, l-Phenylalanine, GGPP, and gibberellins, respectively. Metabolisms in orange, blue, and red backgrounds were amino acid metabolism, synthesis of tanshinones, and synthesis of salvianolic acids, respectively. log2(FC) stands for an estimate of the log2-transformed ratio of the relative content of metabolites in roots of the Cd stress group to that of the control group; R, S, and T stand for an estimate of the log2-transformed ratio of the relative content of metabolites in roots of the 25, 50, and 100 mg kg^−1^ Cd stress group to that of the control group, respectively.

Most of the same differential metabolites identified in all the groups were amino acids and organic acids (Figure 4d). This differed from the previous studies, which adopted different data processing methods [12,19]. The major contributors from these metabolites could take part in the synthesis of salvianolic acids and tanshinones (Figures 4 and 6; Table S3). With the increase in the soil Cd concentration, the relative contents of l-Phe and l-Tyr increased and then decreased significantly (*p* < 0.05) (Figure 5a and b), but relative contents of d-Gln and l-Asp declined and then increased significantly (*p* < 0.05) (Figure 5c and d). The maximum relative contents of l-Phe and l-Tyr are both shown in roots with the treatment of 100 mg kg^−1^ Cd (Figure 5a and b). Besides, with the increase in the soil Cd concentration, the relative content of GGPP decreased and then increased significantly (*p* < 0.05) (Figure 5e), and the relative content of RA increased significantly (*p* < 0.05) (Figure 5f). Besides, GGPP was negatively correlated with l-Tyr and l-Phe, respectively (*p* < 0.05); RA was positively correlated with d-Gln and l-Asp, respectively (*p* < 0.05); l-Asp was positively correlated with d-Gln (*p* < 0.05) (Table 2).

**Table 2:** Correlations (*r*, Pearson’s correlation coefficient) among SOD, CAT, POD, d-Gln, l-Asp, l-Tyr, l-Phe, GGPP, and RA in SM roots

*R*	SOD	CAT	POD	d-Gln	l-Asp	l-Tyr	l-Phe	GGPP	RA
SOD	1								
CAT	−0.03	1							
POD	−0.24	0.26	1						
d-Gln	−0.41	−0.23	−0.32	1					
l-Asp	−0.28	0.34	−0.19	0.79^**^	1				
l-Tyr	0.01	0.77^**^	0.66^*^	−0.58	−0.18	1			
l-Phe	0.26	0.92^**^	0.14	−0.42	0.21	0.74^**^	1		
GGPP	−0.38	−0.59	−0.34	0.88^**^	0.42	**−0.76** ^**^	**−0.77** ^**^	1	
RA	−0.10	0.54	−0.26	**0.61** ^*^	**0.95** ^**^	0.03	0.46	0.20	1

l-Asp, l-Tyr, l-Phe, GGPP, and RA stand for proline, d-glutamine, l-aspartic acid, l-tyrosine, l-phenylalanine, geranylgeranyl-PP, and rosmarinic acid, respectively; significance, *p* value < 0.05: ^*^, *p* < 0.05; ^**^, *p* < 0.01.

## 4 Discussion

### 4.1 Accumulation characteristics of Cd in SM

The study revealed that SM was a non-Cd-hyperaccumulator with a substantial part of Cd enriched in roots. Although in the pot experiment, Cd concentration in leaves was lower than that in roots under each level of soil Cd treatment, the Cd concentration in the two tissues was much smaller than 100 mg kg^−1^ (the threshold value for the Cd-hyperaccumulator in soils is 100 mg kg^−1^ Cd) (Figure 1). TF could reveal the ability to enrich and transport heavy metals from roots to aboveground parts in plants [28]. BCF could reflect the enrichment ability of plants [29]. All the TFs and BCFs in Cd addition groups were below 1 (Table 1). The enrichment characteristics of Cd in SM differed from the Cd-hyperaccumulators, which could enrich at least 100 mg kg^−1^ Cd in soils of 100 mg kg^−1^ Cd or more, with the TF and the seedling BCF of above 1 under different levels of Cd stress [5,32].

Simultaneously, in the study, with the increase in the Cd concentration, change trend of POD and CAT activities, and the proline content in SM were different from those in hyperaccumulator *Phytolacca americana* (Figure 2) [33]. As soil Cd concentration rose, SM absorbed much Cd in roots, part of which was transferred to leaves, resulting in the increase in the leaf Cd concentration (Figure 1a). As soil-added Cd levels increased, the dilution of root Cd was enhanced along with the remarkable increase in TF and BCFs (Table 1; Figure 1) [34]. Similarly, with the increasing concentration of added Cd, Cd concentration in roots and leaves of *Murraya paniculata*, *Catharanthus roseus*, and *Loropetalum chinense* increased, and the roots of all these plants could accumulate more Cd than the leaves [35].

Other Cd was accumulated in roots and root Cd concentration increased. The abiotic stress could cause metabolic disorders which affected cell mass, lipid content, and/or morphological structures [15]. The enhanced Cd would lead to the accumulation of ROS and the peroxidation of membrane lipid in Cd non-hyperaccumulating plants [36,37]. The synergism of antioxidant enzymes (e.g., POD, CAT, and SOD) in plants could eliminate oxygen free radical and alleviate Cd toxicity with the antioxidant enzymes activities significantly increasing under low Cd stress (Figure 2). In many non-Cd-hyperaccumulators, due to low Cd tolerance, the activities of antioxidant enzymes would present a declining trend with the increase in the Cd level [7,12]. However, they might still remarkably clean the active oxygen free radical and lower the lipid peroxidation of plants, resulting in the lower MDA content of plants under Cd stress than that in other Cd-contaminated plants (Figure 2) [7]. The change trends of the antioxidant enzyme activities differed from those of *P. americana*, but showed the same change as *Daucus carota*, under Cd stress conditions [38,39].

### 4.2 Effect of Cd on the synthesis of phenolic acids in SM

Cd stress enhanced the synthesis of phenolic acids (especially RA) in SM roots, which was the precursor of other salvianolic acids with complex structure, theoretically, and boosted with soil Cd increasing (Figure 5) [8,40]. In the study, the major contributors in distinguishing the samples in different levels of Cd stress groups included d-Gln, l-Phe, l-Tyr, and RA (Figure 4; Table S2). First, SM roots could enhance the synthesis of RA indirectly by regulating the metabolism of d-Gln and l-Asp (Figure 6). Amino acids (e.g., Gln, Asp, and Tyr) could participate in the detoxification of plants, such as nitrogen metabolism and secondary metabolism [41,42,43], and bind with the heavy metal ions to reduce their toxicity [14]. Among them, Gln was the first type of organic N compounds converted from N absorbed by plants from soils and participated in various life activities (e.g., amino acid metabolism) [11], and Asp was a free amino acid, and acted as the amino donor of amino acid metabolism, nucleotide metabolism, and TCA cycle [44]. In the current study, l-Asp was positively correlated with d-Gln, and both d-Gln and l-Asp were positively correlated with RA (*p* < 0.05) and, as soil-added Cd concentration increased from 25 to 100 mg kg^−1^, the relative contents of d-Gln and l-Asp increased (Figure 5; Table 2). This might be because as soil Cd concentration increased, the increased d-Gln formed into glutamate, which was a free amino acid, as l-Asp was, and could bind Cd and alleviate toxic effects of Cd on roots (Figure 6) [45]. This might retain the concentration of free Cd in roots to the level to enhance some metabolisms (e.g., RA synthesis) (Figure 6) [6]. By the way, this also suggested that amino group was the main site of Cd binding in SM.

Second, SM roots could enhance the synthesis of RA indirectly by regulating the metabolism of l-Phe and l-Tyr (Figure 6). l-Phe and l-Tyr were the precursors of phenylpropanoid pathway and the tyrosine-derived pathway, respectively, and both the pathways were involved in the biosynthesis of RA [8]. In the study, as soil-added Cd concentration changed from 0 to 50 mg kg^−1^, relative contents of RA, l-Phe, and l-Tyr increased significantly (*p* < 0.05) (Figure 5). However, with soil-added Cd concentration increasing from 50 to 100 mg kg^−1^, the relative content of RA increased significantly and relative contents of l-Phe and l-Tyr declined significantly (*p* < 0.05), with the relative contents of both the amino acids in roots of 100 mg kg^−1^ Cd-added group becoming twice more than that of the control group, respectively (Figure 5). Both POD and CAT showed positive correlations with l-Tyr, and CAT was positively correlated with l-Phe (Table 2). This might be caused by that, although both the relative contents of l-Phe and l-Tyr declined as soil Cd concentration ranged from 50 to 100 mg kg^−1^, the accumulated l-Phe and l-Tyr, coupled with the enhancement of POD and CAT activities, could also promote the synthesis of RA in SM roots (Table 2), which in turn could alleviate Cd toxicity [14]. Conversely, Strejckova et al. [46] and Zhang et al. [19] proved that Cd stress restrained the synthesis of RA in *Scenedesmus quadricauda* and SM, respectively. The differences might be resulted from that the tolerance and response to Cd varied greatly among plants of different species, varieties, or populations [47].

### 4.3 Effect of Cd on the synthesis of tanshinones in SM

As reported above, GGPP was one of the major contributors mainly in distinguishing samples of the control and Cd-added groups (Table S2). GGPP was the critical precursor of synthesizing tanshinones, parts of the main active ingredients of SM roots [8]. In the present study, with increasing soil Cd concentration, the relative content of GGPP significantly decreased and then increased (*p* < 0.05), with the relative content of GGPP in the control group higher than those in the Cd-added groups (Figure 5e). This illustrated that Cd stress could suppress the synthesis of tanshinones. Consistent with the result of the study, Zhang et al. [19] revealed that the accumulation of tanshinones was higher in the control group than that of the Cd-treated group. This might be caused by the comprehensive regulation of metabolites and genes.

On the one hand, the synthesis of tanshinones was inhibited due to the competitive consumption of phosphoenol pyruvate (PEP) in the process of phenolic acid accumulation in SM roots under Cd stress. PEP is an important metabolite connecting glycolysis, tanshinone synthesis, and phenolic acid synthesis (Figure 6) [48]. In the study, GGPP was negatively correlated with both l-Tyr and l-Phe (*p* < 0.05), both of which were closely related to RA synthesis (Figure 6; Table 2) [8]. With high concentration of Cd stress, RA was enriched in SM roots (Figure 5). This would consume a certain amount of PEP, which might lead to the content of PEP being lower than the detectable level and the decrease in GGPP in the study (Table S1). On the other hand, Cd stress suppressed the synthesis of tanshinones via downregulating the expression of GGPPS genes. In the present study, the relative content of GGPP in the control group was higher than those of all the Cd-added groups in SM roots (Figure 5e). The expression of genes in SM roots could be affected by Cd stress [49]. Ali et al. [50] discovered that Cd could downregulate the expression of GGPPS genes, exhibiting a wide range of responses to abiotic stresses, and suppressed the activities of GGPP synthases in SM roots.

## 5 Conclusion

Cd is one of the major heavy metal pollutants in the environment threatening human health by affecting medicinal plant quality. Our study characterized the associated roles of physiological and metabolic regulation in the synthesis of active ingredients in SM with different levels of Cd treatment. First, in all groups, the Cd concentration in leaves was lower than that in roots, and all the TF and BCFs in Cd-treated groups were below 1. Besides, with the increase in the soil Cd concentration, the proline content increased and then declined significantly with the decrease in MDA content; activities of POD and CAT rose and then declined significantly with SOD activity showing no significant differences. This verified that SM was a non-Cd-hyperaccumulator and primarily enriched Cd in roots, with proline, POD, and CAT playing vital roles in resisting Cd stress.

Second, amino acids (especially d-Gln, l-Tyr, l-Asp, and l-Phe) and organic acids (especially GGPP and RA) were major contributors in significantly distinguishing samples with different levels of Cd. As soil Cd concentration increased, the relative content of RA increased significantly, GGPP decreased and then increased significantly with GGPP relative content in control group higher than those in Cd-treated groups, d-Gln and l-Asp declined and then increased, and l-Phe and l-Tyr first increased and then decreased. Besides, RA was positively related to both d-Gln and l-Asp, and GGPP was negatively related to both l-Tyr and l-Phe. This illustrated that Cd treatment enhanced the synthesis of RA by regulating the metabolism of amino acids (especially d-Gln, l-Tyr, l-Asp, and l-Phe) but might suppress the synthesis of tanshinones by decreasing the content of GGPP. The findings revealed Cd enrichment laws of SM and its active ingredient synthesis response to Cd by regulating metabolism and physiology. This provides theoretical basis for the further study on molecular mechanisms of SM response to Cd.

## Supplementary Material

Supplementary Table
