# Supplementary material for: Effects of cadmium on the synthesis of active ingredients in Salvia miltiorrhiza
Source: Open Life Sci. 2023 May 24;18(1):20220603. doi: 10.1515/biol-2022-0603 (PMC10224630; doi:10.1515/biol-2022-0603)
Supplement: Supplementary Table [file biol-2022-0603-sm.pdf]

Supplementary material

Table S1: Metabolites identified based on LC-MS in *S. miltiorrhiza* roots with different levels of soil Cd stress

NO.	Metabolites	RT (s)	Mass	CK		TR		TS		TT	
				Mean	SE	Mean	SE	Mean	SE	Mean	SE
1	N,N'-Diacetylhydrazine	10.7241	115.04	0.47	0.11	0.24	0.02	0.31	0.05	0.81	0.10
2	2,2-Dimethylsuccinic acid	14.8230	145.05	0.03	0.00	0.04	0.00	0.03	0.01	0.07	0.00
3	Ethyl glucuronide	18.3728	221.07	0.05	0.00	0.05	0.01	0.02	0.01	0.10	0.01
4	Succinic acid semialdehyde	23.0758	101.02	0.96	0.28	0.62	0.02	0.41	0.05	1.45	0.13
5	Glucose 6-phosphate	39.4271	259.02	0.55	0.03	0.40	0.01	0.41	0.02	0.68	0.03
6	L-Aspartic acid	40.5494	132.03	4.69	0.06	4.16	0.09	6.33	0.15	7.07	0.21
7	Inosine	40.5496	267.07	0.16	0.01	0.14	0.01	0.19	0.01	0.25	0.01
8	γ-Aminobutyric acid	41.6726	102.06	1.02	0.03	0.64	0.01	1.07	0.05	1.33	0.02
9	L-Glutamic acid	41.6747	146.05	6.88	2.40	6.88	0.06	10.55	0.41	12.64	0.22
10	Gluconic acid	41.6852	195.05	1.13	0.00	0.63	0.01	1.20	0.04	1.15	0.05
11	D-2,3-Dihydroxypropanoic acid	41.6877	105.02	0.29	0.01	0.31	0.06	0.31	0.04	0.41	0.03
12	β-Alanine	42.7915	88.04	0.80	0.01	1.04	0.03	1.33	0.04	0.97	0.03
13	Glycine	42.8068	74.02	0.48	0.03	0.42	0.01	0.65	0.08	1.14	0.06
14	L-Asparagine	42.8099	131.05	6.15	0.08	3.46	0.04	8.03	0.05	6.16	0.07
15	myo-Inositol	43.8189	179.06	4.11	0.02	5.03	0.09	6.11	0.06	5.94	0.14
16	D-Glutamine	43.9210	145.06	56.31	0.68	29.64	0.28	49.66	0.84	71.44	1.28
17	1,3,7-Trimethyluric acid	43.9228	209.07	1.44	0.01	0.79	0.01	1.11	0.04	1.37	0.01
18	benzene-1,2,4-triol	43.9339	125.04	0.15	0.03	0.10	0.02	0.13	0.02	0.21	0.02
19	Gluconolactone	43.9396	177.04	0.44	0.02	0.53	0.02	0.57	0.02	0.79	0.07
20	L-Histidine	43.9413	154.06	0.30	0.01	0.26	0.01	0.62	0.02	0.86	0.04
21	Dihydrolipoate	44.4902	207.05	0.04	0.00	0.04	0.00	0.04	0.01	0.04	0.01
22	L-Lactic acid	45.0416	89.02	2.11	0.41	1.86	0.36	2.00	0.31	3.47	0.19
23	L-Erythrulose	45.0531	119.03	0.66	0.06	0.85	0.04	0.68	0.02	1.61	0.07
24	Pyrrolidonecarboxylic acid	46.1993	128.03	6.41	0.16	3.95	0.41	6.27	0.24	6.17	0.28
25	D-Glucurono-6,3-lactone	47.2963	175.02	0.35	0.01	0.47	0.03	0.44	0.01	0.83	0.05
26	L-Proline	47.3289	114.06	3.81	0.04	3.16	0.04	17.47	1.84	16.88	2.18
27	Oxoadipic acid	48.4517	159.03	0.75	0.08	0.92	0.07	0.72	0.05	1.57	0.21
28	1-Kestose	49.5215	503.16	15.26	0.24	11.66	0.22	10.96	0.15	17.20	0.35
29	Citraconic acid	49.5447	129.02	0.60	0.03	0.74	0.11	0.55	0.16	1.11	0.02
30	Citramalic acid	49.5999	147.03	0.68	0.04	0.21	0.01	0.23	0.01	0.37	0.03
31	But-2-enoic acid	50.5531	85.03	0.14	0.01	0.17	0.01	0.37	0.02	0.46	0.06
32	2-Furoic acid	50.6292	111.01	0.72	0.02	0.67	0.05	0.52	0.01	1.18	0.08
33	5-Aminopentanoic acid	50.7066	116.07	2.43	0.03	4.55	0.12	7.32	0.18	4.66	0.14
34	L-Tyrosine	51.8316	180.07	0.57	0.02	1.57	0.05	2.68	0.10	0.73	0.20
35	Uridine	52.5865	243.06	0.08	0.00	0.07	0.01	0.03	0.01	0.02	0.01
36	Oxytetracycline	52.9640	459.14	0.49	0.06	0.41	0.01	0.37	0.02	0.41	0.05
37	Succinic acid	54.0272	117.02	49.67	0.24	27.46	0.38	27.33	0.36	49.00	1.96

(*Continued*)

Table S1: *Continued*

NO.	Metabolites	RT (s)	Mass	CK		TR		TS		TT	
				Mean	SE	Mean	SE	Mean	SE	Mean	SE
38	Succinic anhydride	54.0943	99.01	2.30	0.02	1.69	0.04	1.18	0.11	3.05	0.22
39	Mannitol	54.0943	181.07	0.14	0.01	0.14	0.01	0.24	0.00	0.06	0.01
40	L-Serine	59.8012	104.03	0.05	0.00	0.07	0.01	0.09	0.00	0.16	0.03
41	Sucrose	60.8393	341.11	5.96	0.76	7.02	0.55	7.60	0.68	4.24	2.02
42	Theophylline	68.3698	179.06	0.26	0.11	0.32	0.02	0.29	0.02	0.37	0.04
43	Maltopentaose	71.3485	827.27	0.39	0.05	0.73	0.02	0.33	0.02	0.14	0.06
44	D-Tartaric acid	71.8595	149.01	0.05	0.01	0.06	0.00	0.06	0.01	0.09	0.00
45	Fumaric acid	72.2048	115.00	0.52	0.02	0.85	0.13	0.49	0.02	0.90	0.02
46	Stachyose	72.3970	665.22	15.40	1.68	28.39	0.32	9.33	0.76	0.51	0.18
47	Methylsuccinic acid	72.4188	131.03	0.37	0.08	0.39	0.12	0.19	0.01	0.46	0.04
48	L-Gulonolactone	73.4586	177.04	0.22	0.01	0.29	0.08	0.20	0.01	0.38	0.02
49	Raffinose	75.0982	503.16	2.30	0.20	3.58	0.11	1.98	0.17	1.05	0.91
50	Syringic acid	78.1883	197.05	0.69	0.04	1.23	0.05	2.19	0.16	1.40	0.68
51	Pyridoxal	79.0455	166.05	0.08	0.01	0.04	0.01	0.05	0.01	0.04	0.01
52	L-Norleucine	83.5828	130.09	3.62	0.13	5.23	0.13	6.74	0.24	4.64	0.12
53	Itaconic acid	84.0399	129.02	0.24	0.01	0.26	0.01	0.15	0.01	0.37	0.03
54	(2R)-6,8-Diglucopyranosyl-4',5,7-trihydroxyflavanone	85.0693	595.17	0.01	0.00	0.09	0.08	0.02	0.00	0.02	0.01
55	DL-Dopa	85.5300	196.06	0.15	0.00	0.37	0.01	0.76	0.00	0.29	0.08
56	Acetaminophen	85.7146	150.06	0.04	0.00	0.11	0.00	0.20	0.01	0.09	0.02
57	α-Ketoisovaleric acid	91.2160	115.04	0.30	0.00	0.35	0.01	0.11	0.04	0.55	0.16
58	Vanillin	99.4340	151.04	0.20	0.00	0.26	0.00	0.28	0.02	0.42	0.06
59	Trehalose	149.7160	341.11	0.02	0.00	0.02	0.00	0.01	0.00	0.02	0.01
60	Erythrono-1,4-lactone	153.2710	117.02	2.31	0.04	3.24	0.65	2.95	0.23	6.56	0.52
61	Adenine	160.2620	134.05	1.30	0.38	0.63	0.20	0.37	0.16	0.27	0.05
62	Guanosine	187.4840	282.08	0.04	0.00	0.03	0.00	0.02	0.00	0.02	0.01
63	o-Tyrosine	190.6770	180.07	0.15	0.00	0.47	0.01	0.57	0.02	0.31	0.01
64	Betamipron	190.6770	192.07	0.02	0.00	0.05	0.00	0.06	0.00	0.04	0.00
65	L-Phenylalanine	190.6785	164.07	5.79	0.06	17.21	0.44	18.57	0.22	13.37	0.12
66	Pantothenic acid	191.4645	218.10	0.39	0.01	0.46	0.01	0.55	0.01	0.11	0.04
67	N1-(2-Hydroxyethyl)flurazepam	192.8875	331.07	0.01	0.00	0.02	0.00	0.02	0.00	0.02	0.00
68	Imidazoleacetic acid	194.1700	125.04	0.19	0.00	0.25	0.01	0.29	0.01	0.47	0.01
69	L-Arginine	205.9810	173.10	0.03	0.00	0.03	0.00	0.11	0.00	0.06	0.01
70	2',4',6'-Trihydroxyacetophenone	218.0615	167.03	0.15	0.01	0.21	0.01	0.18	0.01	0.20	0.01
71	Terephthalic acid	222.2230	165.02	0.27	0.01	0.31	0.04	0.37	0.01	0.68	0.20
72	D-Malic acid	223.3700	133.01	0.59	0.00	0.65	0.03	0.73	0.04	0.95	0.03
73	Phenobarbital	223.4740	231.08	0.01	0.00	0.01	0.00	0.03	0.00	0.02	0.00
74	5-Hydroxy-L-tryptophan	223.6080	219.08	0.04	0.00	0.06	0.00	0.17	0.01	0.08	0.01
75	Chorismate	225.2405	225.04	0.04	0.01	0.12	0.00	0.30	0.01	0.12	0.01

(*Continued*)

Table S1: *Continued*

NO.	Metabolites	RT (s)	Mass	CK		TR		TS		TT	
				Mean	SE	Mean	SE	Mean	SE	Mean	SE
76	Estrone sulfate	229.4680	349.11	0.03	0.00	0.02	0.00	0.07	0.00	0.04	0.00
77	Phenylethyl primeveroside	229.9560	461.17	0.01	0.00	0.01	0.00	0.00	0.00	0.01	0.00
78	γ-Glutamylcysteine	231.3570	249.05	0.02	0.00	0.04	0.00	0.04	0.00	0.04	0.00
79	3,4-Dihydroxyhydrocinnamic acid	231.5800	181.05	0.53	0.01	0.77	0.01	0.76	0.02	0.94	0.01
80	3-(3,4-Dihydroxy-5-methoxy)-2-propenoic acid	232.6030	209.05	0.40	0.01	0.49	0.01	0.39	0.01	0.99	0.01
81	Dopamine	234.7840	299.08	0.06	0.00	0.04	0.00	0.09	0.01	0.08	0.01
82	Apiin	237.0730	563.14	0.00	0.00	0.01	0.00	0.01	0.00	0.01	0.00
83	(Ã,Â±)-Tryptophan	240.3660	203.08	0.02	0.01	0.03	0.01	0.27	0.05	0.07	0.02
84	Glucobrassicin	240.4155	447.06	0.02	0.00	0.04	0.00	0.02	0.01	0.02	0.01
85	Trans-3-coumarate	241.2045	163.04	0.07	0.01	0.13	0.01	0.13	0.03	0.09	0.01
86	5,7-dihydroxy-2-(4-methoxyphenyl)-3,4-dihydro-2H-1-benzopyran-4-one	242.7500	285.08	0.04	0.00	0.02	0.00	0.01	0.00	0.01	0.00
87	p-Anisic acid	245.5660	151.04	0.41	0.03	0.56	0.02	0.58	0.01	0.73	0.01
88	3,4-Dihydroxy-trans-cinnamate	246.5080	179.03	8.84	0.09	9.76	0.06	14.08	0.18	18.59	0.19
89	3-Carboxy-4-methyl-5-propyl-2-furanpropionic acid	246.7470	239.09	0.03	0.00	0.02	0.00	0.02	0.00	0.03	0.00
90	Fertaric acid	247.5590	325.06	0.03	0.01	0.03	0.01	0.03	0.00	0.04	0.01
91	3-(4-hydroxy-3-methoxyphenyl)prop-2-enoic acid	247.7055	193.05	0.24	0.04	0.36	0.01	0.48	0.01	0.33	0.01
92	3-Methoxyanthranilate	249.9430	166.05	0.35	0.00	0.41	0.01	0.32	0.01	0.29	0.01
93	Quercetin 3-O-(6''-acetyl-glucoside)	253.5360	505.10	0.03	0.00	0.01	0.00	0.03	0.00	0.00	0.00
94	Caffeic acid	260.4550	179.03	2.32	0.04	3.12	0.09	4.56	0.08	7.09	0.18
95	2-Methylbenzoic acid	260.4550	135.04	2.39	0.07	3.60	0.11	4.44	0.48	7.38	0.06
96	Rosmarinic acid	264.9790	359.08	10.38	0.39	12.47	0.08	35.93	1.26	62.23	2.28
97	Umbelliferone	264.9800	161.02	2.32	0.05	2.58	0.05	7.38	0.14	13.56	0.09
98	Aesculetin	265.0220	177.02	0.08	0.01	0.12	0.01	0.15	0.00	0.23	0.07
99	1,11-Undecanedicarboxylic acid	265.8720	243.16	0.03	0.00	0.02	0.00	0.04	0.00	0.01	0.01
100	Hydroxyphenyllactic acid	267.3150	181.05	0.25	0.01	0.38	0.00	0.49	0.01	0.63	0.01
101	(R)-Kawain	274.1690	229.09	0.02	0.00	0.01	0.00	0.01	0.00	0.02	0.00
102	1,2,3-Trihydroxybenzene	275.4195	125.02	0.14	0.01	0.11	0.01	0.10	0.01	0.20	0.00
103	Eupatilin	278.8890	343.08	0.14	0.00	0.06	0.00	0.16	0.00	0.19	0.01
104	5-Methoxysalicylic acid	284.8570	167.03	4.78	0.14	11.40	0.16	8.97	0.23	12.47	0.12
105	Theaflavin	284.9650	563.12	0.00	0.00	0.01	0.00	0.00	0.00	0.00	0.00
106	(R)-2-Benzylsuccinate	285.9225	207.07	0.08	0.01	0.07	0.01	0.07	0.00	0.05	0.00
107	Xanthoxylin	287.2465	195.07	0.02	0.00	0.02	0.00	0.03	0.00	0.07	0.00
108	Isoferulic acid	289.4125	193.05	0.10	0.00	0.07	0.01	0.09	0.00	0.08	0.01
109	Eudesmic acid	294.0770	211.06	0.19	0.01	0.55	0.01	0.54	0.02	1.56	0.01
110	Enterodiol	296.6410	301.14	0.03	0.01	0.01	0.00	0.04	0.00	0.02	0.00

(*Continued*)

Table S1: *Continued*

NO.	Metabolites	RT (s)	Mass	CK		TR		TS		TT	
				Mean	SE	Mean	SE	Mean	SE	Mean	SE
111	Genipin	298.2050	225.08	0.02	0.00	0.06	0.00	0.03	0.01	0.04	0.00
112	Glycitein	299.0045	283.06	0.02	0.00	0.01	0.00	0.03	0.00	0.01	0.00
113	Aspalathin	299.7360	451.13	0.00	0.00	0.00	0.00	0.01	0.00	0.01	0.00
114	Azelaic acid	304.3050	187.10	0.10	0.00	0.10	0.01	0.08	0.00	0.19	0.01
115	5,7-dihydroxy-2-(4-hydroxyphenyl)-6,8-dimethyl-3,4-dihydro-2H-1-benzopyran-4-one	306.6300	299.09	0.05	0.00	0.02	0.00	0.05	0.00	0.01	0.00
116	Byssochlamic acid	311.4040	331.12	0.10	0.01	0.04	0.01	0.09	0.01	0.07	0.01
117	Methyl vanillate	312.2720	181.05	0.06	0.00	0.05	0.01	0.06	0.00	0.08	0.00
118	γ-CEHC	314.9725	263.13	0.03	0.00	0.01	0.00	0.03	0.00	0.02	0.00
119	Geranylgeranyl-PP	315.0070	449.18	0.03	0.00	0.00	0.00	0.01	0.00	0.02	0.00
120	Glaucarubin	317.2550	495.22	0.01	0.00	0.01	0.00	0.01	0.00	0.01	0.00
121	Meconine	321.9640	193.05	0.03	0.01	0.06	0.00	0.04	0.00	0.06	0.00
122	Benazeprilat	325.3360	395.16	0.14	0.00	0.01	0.00	0.07	0.01	0.03	0.00
123	Corticosterone	330.0290	381.18	0.05	0.01	0.00	0.00	0.02	0.00	0.01	0.00
124	Gibberellin A3	331.2010	345.14	0.09	0.00	0.03	0.00	0.09	0.00	0.05	0.00
125	Aflatoxin B1	332.3690	311.06	0.04	0.00	0.03	0.00	0.03	0.00	0.02	0.00
126	trans-Piceid	335.4460	403.14	0.03	0.00	0.03	0.00	0.04	0.00	0.07	0.00
127	Ascochitine	340.3810	275.09	0.04	0.00	0.02	0.00	0.04	0.00	0.01	0.00
128	Glycitin	345.7380	445.11	0.01	0.00	0.01	0.00	0.01	0.00	0.02	0.00
129	Monoethyl phthalate	347.2630	193.05	1.38	0.02	1.16	0.02	1.78	0.06	2.06	0.01
130	Citicoline	347.2640	487.10	0.00	0.00	0.00	0.00	0.00	0.00	0.00	0.00
131	5,7-dihydroxy-2-(4-hydroxy-3,5-dimethoxyphenyl)-4H-chromen-4-one	348.4230	329.07	0.06	0.01	0.08	0.01	0.09	0.01	0.05	0.00
132	Traumatic acid	351.8855	227.13	0.03	0.01	0.04	0.01	0.04	0.00	0.07	0.01
133	Myricetin 3-neohesperidoside	353.0460	625.14	0.02	0.00	0.02	0.00	0.02	0.00	0.04	0.00
134	Propionic acid	353.8485	73.03	0.24	0.02	0.34	0.10	0.24	0.01	0.53	0.01
135	Malic acid	357.3810	133.01	0.40	0.03	0.40	0.01	0.25	0.00	0.85	0.22
136	Glycocholic acid	360.9280	464.30	0.03	0.00	0.03	0.00	0.03	0.00	0.03	0.00
137	Gibberellin A19	361.1740	361.17	0.07	0.00	0.02	0.00	0.09	0.00	0.03	0.00
138	(S,E)-Zearalenone	361.1810	317.14	0.03	0.00	0.01	0.00	0.02	0.01	0.04	0.01
139	Docosahexaenoic acid	373.8675	327.23	0.15	0.04	0.04	0.01	0.13	0.01	0.05	0.00
140	3-Hydroxycapric acid	374.5355	187.13	0.31	0.02	0.29	0.04	0.36	0.01	0.68	0.04
141	3-Hydroxyflavone	383.3590	237.06	0.13	0.00	0.06	0.00	0.16	0.01	0.07	0.00
142	Citric acid	387.8450	191.02	0.64	0.02	0.46	0.02	0.48	0.05	0.66	0.01
143	Methyl 2-hydroxybenzoate	392.5040	151.04	0.45	0.01	0.43	0.01	0.52	0.02	0.88	0.03
144	Indoleacetaldehyde	392.5070	158.06	2.04	0.05	3.15	0.02	7.01	0.04	3.27	0.04
145	bicyclo-PGE2	397.1400	333.21	0.04	0.00	0.01	0.00	0.03	0.00	0.02	0.00
146	Mono-benzyl phthalate	398.0830	255.07	0.02	0.00	0.00	0.00	0.03	0.00	0.04	0.00

(*Continued*)

Table S1: *Continued*

NO.	Metabolites	RT (s)	Mass	CK		TR		TS		TT	
				Mean	SE	Mean	SE	Mean	SE	Mean	SE
147	Levocetirizine	401.7090	387.15	0.02	0.00	0.00	0.00	0.01	0.00	0.01	0.00
148	Heptanoic acid	402.8625	129.09	0.23	0.01	0.25	0.00	0.26	0.01	0.50	0.02
149	8-iso-15-keto-PGE2	407.4190	349.20	0.04	0.00	0.01	0.00	0.02	0.00	0.01	0.00
150	Vanillic acid	410.0320	167.03	0.04	0.01	0.06	0.01	0.04	0.00	0.10	0.02
151	Leukotriene B4	413.8660	335.22	0.04	0.01	0.01	0.00	0.03	0.00	0.01	0.00
152	6β-Hydroxyasiatic acid	414.6740	503.34	0.24	0.00	0.04	0.00	0.15	0.00	0.09	0.00
153	Daidzin	415.7995	461.11	0.00	0.00	0.00	0.00	0.00	0.00	0.01	0.00
154	Mycophenolic acid	419.3375	319.12	0.03	0.00	0.01	0.00	0.02	0.00	0.00	0.00
155	Cohumulone	421.6130	347.19	0.71	0.01	0.26	0.00	0.54	0.08	0.38	0.01
156	[6]-Dehydrogingerdione	428.8840	289.15	0.01	0.00	0.01	0.00	0.03	0.00	0.01	0.00
157	Formononetin	430.0605	267.07	0.05	0.00	0.02	0.00	0.03	0.00	0.02	0.00
158	Caprylic acid	430.6780	143.11	0.52	0.04	0.52	0.01	0.52	0.01	1.03	0.03
159	[8]-Dehydrogingerdione	433.4030	317.18	0.04	0.00	0.02	0.00	0.03	0.01	0.04	0.00
160	Demethoxycurcumin	435.2040	337.11	0.00	0.00	0.00	0.00	0.00	0.00	0.01	0.00
161	Butylparaben	439.9290	193.09	0.01	0.00	0.02	0.00	0.01	0.00	0.03	0.00
162	Gingerol	440.5970	293.18	0.44	0.01	0.57	0.01	0.40	0.01	0.65	0.01
163	Isokobusone	441.0850	221.15	0.15	0.01	0.19	0.00	0.15	0.01	0.21	0.00
164	Secoisolariciresinol	443.0875	361.17	0.06	0.01	0.01	0.00	0.07	0.01	0.05	0.00
165	Gibberellin A44	443.7450	345.17	0.40	0.02	0.11	0.00	0.30	0.02	0.20	0.00
166	4-hydroxy-3-[1-(4-hydroxyphenyl)-3-oxobutyl]-2H-chromen-2-one	445.5930	323.09	0.08	0.00	0.05	0.00	0.05	0.00	0.03	0.00
167	(-)-Matairesinol	445.9630	357.13	0.27	0.02	0.14	0.00	0.14	0.01	0.18	0.01
168	4-[(E)-2-(3,5-dimethoxyphenyl)ethenyl] phenol	449.9785	255.10	0.15	0.01	0.03	0.01	0.15	0.01	0.03	0.00
169	Methyl jasmonate	450.7100	223.13	0.01	0.00	0.01	0.00	0.02	0.00	0.02	0.00
170	2-Hydroxyestradiol	454.0490	287.17	0.28	0.01	0.06	0.00	0.42	0.01	0.37	0.01
171	Norbuprenorphine	455.3365	412.25	0.02	0.01	0.01	0.00	0.01	0.00	0.00	0.01
172	Medicagenic acid	458.6755	501.32	0.04	0.01	0.01	0.00	0.07	0.00	0.05	0.00
173	(R)-3-Hydroxy-tetradecanoic acid	458.6940	243.20	0.15	0.01	0.18	0.01	0.13	0.01	0.37	0.08
174	Prostaglandin D3	463.2940	349.20	0.05	0.00	0.01	0.00	0.04	0.01	0.02	0.00
175	[4]-Gingerdiol 3,5-diacetate	464.7375	351.18	0.03	0.01	0.00	0.00	0.04	0.00	0.00	0.00
176	Methylgingerol	466.2470	307.19	0.04	0.00	0.00	0.00	0.04	0.00	0.01	0.00
177	8-iso-PGA1	469.0025	335.22	0.04	0.01	0.01	0.00	0.05	0.00	0.02	0.00
178	Leukotriene B5	472.6090	333.21	3.79	0.08	0.87	0.01	2.51	0.12	0.80	0.06
179	Resolvin D2	473.9210	375.22	0.01	0.00	0.01	0.00	0.08	0.01	0.01	0.00
180	15-Deoxy-d-12,14-PGJ2	475.0145	315.20	3.40	0.06	0.75	0.02	2.22	0.06	0.72	0.05
181	Pelargonic acid	475.9910	157.12	2.98	0.01	2.89	0.04	2.92	0.05	4.11	0.04
182	Tetrahydrocurcumin	476.1150	371.15	0.05	0.01	0.02	0.00	0.14	0.00	0.05	0.00
183	α-Linolenic acid	480.0740	277.22	0.05	0.00	0.00	0.00	0.02	0.00	0.05	0.00

(*Continued*)

Table S1: *Continued*

NO.	Metabolites	RT (s)	Mass	CK		TR		TS		TT	
				Mean	SE	Mean	SE	Mean	SE	Mean	SE
184	4',5,7-Trihydroxy-6-prenylflavanone	482.0050	339.12	0.11	0.00	0.06	0.01	0.08	0.00	0.04	0.00
185	Cortisone	483.6220	359.19	0.17	0.03	0.05	0.00	0.08	0.01	0.11	0.00
186	Dihydrojasmonic acid	485.8090	211.13	0.53	0.01	0.58	0.01	0.57	0.01	1.37	0.02
187	Gibberellin A53	489.2330	347.19	0.38	0.08	0.07	0.01	0.29	0.01	0.13	0.00
188	8,15-DiHETE	490.5355	335.22	0.04	0.00	0.05	0.00	0.08	0.01	0.04	0.01
189	Ethyl hexadecanoate	495.8925	283.26	0.09	0.02	0.11	0.00	0.01	0.00	0.31	0.04
190	Capric acid	496.1070	171.14	0.66	0.12	0.50	0.07	0.46	0.01	0.81	0.03
191	20-Hydroxyeicosatetraenoic acid	496.1255	319.23	0.51	0.01	0.23	0.00	0.94	0.01	0.32	0.00
192	6β-Hydroxytestosterone	500.4950	303.20	0.11	0.01	0.02	0.00	0.10	0.00	0.03	0.00
193	Linoleic acid	502.5945	279.23	0.19	0.01	0.01	0.00	0.07	0.00	0.18	0.00
194	4-Dodecylbenzenesulfonic Acid	504.9300	325.18	0.42	0.01	0.45	0.01	0.39	0.01	0.64	0.06
195	Estriol	506.4725	287.17	0.76	0.02	0.15	0.00	0.45	0.01	0.66	0.02
196	Resolvin D1	507.6850	375.22	0.17	0.00	0.01	0.00	0.06	0.00	0.06	0.00
197	Geranylacetone	509.2375	329.29	0.07	0.01	0.04	0.01	0.03	0.01	0.05	0.00
198	Thyrotropin releasing hormone	510.6950	361.17	0.05	0.01	0.02	0.00	0.04	0.01	0.05	0.00
199	LysoPA(16:0/0:0)	512.1710	409.24	0.03	0.00	0.01	0.01	0.02	0.02	0.01	0.00
200	Esculentic acid (Diplazium)	514.0910	487.34	7.05	0.08	1.26	0.02	3.55	0.06	5.12	0.12
201	11-Dehydrocorticosterone	518.9910	343.19	0.11	0.01	0.02	0.00	0.09	0.01	0.04	0.00
202	Estradiol	520.1230	271.17	13.62	0.08	1.95	0.04	8.32	0.06	14.98	0.26
203	5-HEPE	523.3710	317.21	3.68	0.05	0.89	0.01	2.08	0.09	1.01	0.08
204	2',5,6-Trimethoxyflavone	524.4140	311.09	0.10	0.01	0.05	0.01	0.09	0.01	0.08	0.01
205	11(R)-HETE	524.6510	319.23	0.15	0.00	0.13	0.00	0.23	0.01	0.11	0.01
206	Cortisol	526.6860	361.20	0.70	0.03	0.10	0.00	0.44	0.09	0.31	0.01
207	[10]-Dehydrogingerdione	529.0950	345.21	3.76	0.11	0.52	0.00	1.97	0.19	1.22	0.04
208	Ethyl dodecanoate	532.6140	227.20	0.05	0.00	0.07	0.01	0.06	0.00	0.14	0.00
209	(10E,12Z)-9-HODE	534.7250	295.23	0.40	0.01	0.05	0.00	0.09	0.01	0.10	0.00
210	Undecanoic acid	540.5345	185.15	0.69	0.01	0.72	0.02	0.76	0.01	1.47	0.04
211	Carnosol	545.7535	329.18	0.85	0.01	0.43	0.01	1.29	0.15	0.73	0.04
212	1,7-bis(4-hydroxyphenyl)-5-methoxyheptan-3-one	546.3315	327.16	0.52	0.01	0.31	0.01	0.23	0.02	0.26	0.00
213	(2E)-3-(2-hydroxyphenyl)-1-(4-methoxyphenyl)prop-2-en-1-one	547.4480	253.09	0.03	0.00	0.01	0.00	0.02	0.00	0.02	0.00
214	Dehydroepiandrosterone	547.4790	287.20	0.21	0.01	0.05	0.01	0.17	0.00	0.07	0.01
215	LysoPE(18:1(9Z)/0:0)	549.7480	478.29	0.02	0.01	0.01	0.00	0.00	0.00	0.01	0.00
216	18R-HEPE	550.8900	317.20	2.88	0.04	0.63	0.16	2.27	0.05	1.06	0.32
217	13-OxoODE	552.0260	293.21	0.23	0.01	0.03	0.01	0.15	0.00	0.07	0.01
218	Tridecanoic acid	552.5855	213.19	0.11	0.01	0.13	0.02	0.12	0.01	0.26	0.04
219	9-cis-Retinoic acid	553.1475	299.20	73.25	0.17	27.71	0.42	66.46	1.43	49.43	0.39
220	L-Malic acid	558.8125	133.01	1.01	0.04	0.77	0.02	0.51	0.00	1.15	0.01

(*Continued*)

Table S1: *Continued*

NO.	Metabolites	RT (s)	Mass	CK		TR		TS		TT	
				Mean	SE	Mean	SE	Mean	SE	Mean	SE
221	2-Methoxyestrone	558.8540	299.17	1.92	0.06	0.80	0.01	1.55	0.19	1.82	0.20
222	16-Hydroxy hexadecanoic acid	558.8590	271.23	0.09	0.02	0.11	0.02	0.12	0.01	0.19	0.01
223	Estrone	559.9470	269.15	0.34	0.01	0.09	0.01	0.15	0.01	0.23	0.02
224	Palmitic acid	562.2520	255.23	0.13	0.00	0.15	0.01	0.09	0.01	0.34	0.01
225	all-trans-Retinoic acid	567.8640	299.20	3.11	0.14	1.39	0.01	3.07	0.25	2.69	0.17
226	Dodecanoic acid	570.1730	199.17	0.92	0.04	1.09	0.01	1.13	0.02	2.04	0.05
227	Steviobioside	575.3325	641.31	0.02	0.00	0.00	0.00	0.02	0.00	0.01	0.00
228	γ-Glutamylmethionine	575.9740	277.09	1.63	0.07	0.94	0.07	1.85	0.10	0.78	0.01
229	Eicosapentaenoic acid	579.3335	301.22	5.46	0.26	2.57	0.06	5.11	0.02	5.67	0.08
230	Isopalmitic acid	579.8880	255.23	0.11	0.01	0.13	0.00	0.08	0.00	0.29	0.01
231	12-HEPE	581.4510	317.21	1.16	0.01	0.48	0.01	1.17	0.04	1.02	0.04
232	2,3-Dihydroxybutanedioic acid	581.5135	149.01	1.55	0.04	1.63	0.01	1.52	0.05	3.11	0.05
233	Myristoleic acid	589.8555	225.19	0.05	0.00	0.05	0.01	0.07	0.01	0.08	0.01
234	Carnosic acid	590.6010	331.19	2.03	0.05	0.40	0.01	2.32	0.05	0.73	0.01
235	Maslinic acid	595.1580	471.35	0.67	0.01	0.17	0.00	0.23	0.01	0.71	0.02
236	Arachidonic acid	597.4330	303.23	0.04	0.00	0.02	0.00	0.04	0.00	0.03	0.00
237	2-Ketobutyric acid	599.6950	101.02	0.41	0.08	0.33	0.01	0.17	0.02	0.65	0.07
238	21-Hydroxypregnenolone	605.3220	331.23	17.72	0.10	2.15	0.09	11.35	0.59	1.87	0.03
239	Dehydroabietic acid	605.6125	299.20	8.66	1.54	1.90	0.03	5.47	0.12	3.19	0.18
240	Cholesterol sulfate	609.4680	465.31	0.04	0.01	0.01	0.00	0.01	0.01	0.02	0.00
241	15-KETE	609.8380	317.21	104.88	1.78	4.97	0.09	35.72	0.91	7.89	0.48
242	Ethyl stearate	610.4305	311.30	0.02	0.01	0.02	0.01	0.04	0.01	0.07	0.04
243	3α-Acetoxy-11-keto-β-boswellic acid	611.6110	511.34	0.01	0.01	0.00	0.00	0.02	0.00	0.00	0.00
244	Glycyrrhetinic acid	620.0490	469.33	3.25	0.06	0.70	0.01	1.49	0.02	1.84	0.01
245	2-Methoxyestradiol	622.9190	301.18	0.71	0.05	0.62	0.06	0.41	0.14	1.53	0.29
246	Suberic acid	623.1150	173.08	0.25	0.01	0.36	0.02	0.28	0.02	0.73	0.02
247	Pimelic acid	623.1150	159.07	0.94	0.13	1.06	0.16	0.74	0.09	3.08	0.88
248	Myristic acid	624.5770	227.20	1.24	0.03	1.10	0.04	1.31	0.04	2.14	0.09
249	3,4-Dihydroxyphenylglycol	625.7210	169.05	0.22	0.05	0.35	0.13	0.14	0.02	0.56	0.21
250	Adipic acid	625.7730	145.05	4.39	0.88	3.30	0.84	2.25	0.28	8.86	3.55
251	Hypogeic acid	638.0265	253.22	0.84	0.21	0.43	0.05	0.49	0.04	1.12	0.17
252	16(17)-EpDPE	639.2690	343.23	0.06	0.01	0.20	0.14	0.72	0.61	0.01	0.01
253	Epiandrosterone	640.4580	289.22	0.26	0.01	0.10	0.01	0.20	0.01	0.07	0.01
254	Pentadecanoic acid	641.5060	241.22	0.29	0.01	0.27	0.01	0.27	0.01	0.52	0.02
255	Bovinic acid	644.9405	279.23	10.23	0.21	1.50	0.05	2.79	0.06	4.71	0.09
256	Ursolic acid	644.9410	455.35	8.69	0.36	2.42	0.08	4.37	0.12	9.80	0.25
257	Cytosine	651.6490	110.04	1.82	0.51	1.12	0.18	0.88	0.05	2.08	0.08
258	**Glycyltyrosine**	651.6835	237.09	1.00	0.15	0.98	0.36	0.95	0.09	2.64	1.02
259	Threonic acid	657.3620	135.03	2.00	0.87	0.91	0.06	1.21	0.02	3.54	1.26

(*Continued*)

Table S1: *Continued*

NO.	Metabolites	RT (s)	Mass	CK		TR		TS		TT	
				Mean	SE	Mean	SE	Mean	SE	Mean	SE
260	3-Methyladipic acid	659.0490	159.07	3.46	0.41	8.20	2.97	2.95	0.11	6.57	0.63
261	2-Isopropylmalic acid	665.2770	175.06	1.26	0.24	1.75	0.48	1.15	0.19	3.12	0.92
262	cis,cis-Muconic acid	669.7900	141.02	1.47	0.21	1.73	0.51	3.52	1.03	2.70	0.54
263	Ethyl tetradecanoate	669.7930	255.23	12.73	0.28	6.28	0.03	8.40	0.28	12.55	0.75
264	Oleic acid	676.5640	281.25	4.07	0.14	0.78	0.02	1.57	0.06	1.97	0.07
265	Salicyluric acid	678.8300	194.05	0.27	0.01	0.86	0.22	0.52	0.06	0.83	0.12
266	Porphobilinogen	681.6780	225.09	3.19	0.49	5.24	1.07	6.37	3.19	6.97	0.68
267	Uracil	690.6650	111.02	0.98	0.17	1.37	0.19	1.40	0.08	2.96	1.11
268	15-Methylpalmitate	693.4880	269.25	0.22	0.01	0.09	0.01	0.24	0.01	0.23	0.03
269	Allocystathionine	697.9965	221.06	0.45	0.04	0.47	0.02	0.57	0.07	1.13	0.09
270	Succinylacetone	713.7990	157.05	9.12	0.90	13.70	1.93	15.37	1.79	29.41	6.22
271	Kojic acid	730.7360	141.02	7.45	2.75	5.39	0.12	6.54	0.39	18.99	2.71
272	Hypoxanthine	742.0350	135.03	2.38	0.99	2.00	0.11	1.86	0.06	6.09	0.74
273	2,6-Pyridinedicarboxylic acid	743.1530	166.02	0.94	0.04	1.09	0.24	1.59	0.26	3.84	0.72
274	2-Furoylglycine	746.4990	168.03	1.83	0.34	2.75	0.74	2.31	0.25	4.61	0.69
275	4-Pyridoxic acid	761.1675	182.05	4.58	0.63	7.85	1.39	7.83	0.64	15.63	2.01
276	Methyldopa	769.0995	210.08	5.28	2.16	5.82	1.92	5.43	1.57	11.67	3.35
277	2-Methylglutaric acid	769.1300	145.05	99.76	38.39	105.63	30.31	71.49	31.66	222.10	92.49
278	D-Xylulose	776.8875	149.05	0.88	0.19	2.23	0.56	3.14	0.74	1.91	0.16
279	5-Methylcytosine	779.2965	124.05	1.51	0.02	1.63	0.10	2.43	0.61	3.90	0.18
280	Acetylisoniazid	780.3070	178.06	0.13	0.01	0.13	0.01	0.16	0.00	0.30	0.02
281	Pyruvic acid	780.4235	87.01	0.86	0.01	0.88	0.04	0.68	0.04	1.89	0.11
282	3-Furoic acid	784.8590	111.01	0.31	0.01	0.36	0.02	0.23	0.01	0.60	0.04
283	Gallic acid	784.8590	169.01	0.22	0.00	0.29	0.02	0.12	0.01	0.59	0.03
284	Phenylglyoxylic acid	786.1190	149.02	0.17	0.03	0.21	0.04	0.13	0.01	0.37	0.04
285	(R)-lipoic acid	786.7375	223.02	0.07	0.01	0.08	0.01	0.10	0.02	0.20	0.01
286	3-Hydroxypicolinic acid	793.1100	138.02	0.48	0.16	0.43	0.01	0.15	0.03	0.55	0.22
287	Stearic acid	793.6710	283.26	0.04	0.01	0.04	0.00	0.01	0.00	0.11	0.01
288	Pyroglutamic acid	795.4360	128.03	0.56	0.08	0.66	0.04	0.31	0.15	1.51	0.55
289	2-Oxovaleric acid	796.5930	115.04	0.40	0.14	0.42	0.15	0.37	0.04	0.53	0.09
290	M-toluic Acid	798.9830	135.04	0.26	0.03	0.31	0.02	0.21	0.04	0.39	0.06
291	Glyceraldehyde	804.2610	89.02	0.54	0.02	0.70	0.06	1.33	0.06	3.27	2.29
292	Quinic acid	809.1910	191.06	0.04	0.00	0.05	0.00	0.04	0.01	0.10	0.00
293	3-Hydroxymethylglutaric acid	809.5170	161.05	0.37	0.09	0.23	0.04	0.22	0.04	0.46	0.11
294	6-Hydroxynicotinic acid	820.5530	138.02	0.32	0.02	0.31	0.02	0.19	0.01	0.74	0.16
295	3-Hydroxybenzoic acid	833.2770	137.02	0.27	0.06	0.25	0.03	0.13	0.01	0.41	0.03
296	3-Methyl-2-oxovaleric acid	840.6390	129.06	0.17	0.01	0.22	0.01	0.14	0.01	0.43	0.03
297	Nicotinic acid	842.0860	122.02	1.67	0.28	2.08	0.31	1.57	0.12	3.59	0.65
298	Phenylacetic acid	842.1030	135.04	0.20	0.03	0.23	0.05	0.14	0.01	0.50	0.07

(*Continued*)

Table S1: *Continued*

NO.	Metabolites	RT (s)	Mass	CK		TR		TS		TT	
				Mean	SE	Mean	SE	Mean	SE	Mean	SE
299	Glutaric acid	843.0880	131.03	0.11	0.02	0.11	0.01	0.09	0.00	0.19	0.01
300	Pyrrole-2-carboxylic acid	856.8650	110.02	0.26	0.02	0.24	0.03	0.21	0.02	0.44	0.03
301	3-Hydroxyphenylacetic acid	864.2995	151.04	0.19	0.07	0.17	0.05	0.09	0.00	0.27	0.01
302	Dimethylmalonic acid	870.4745	131.03	0.17	0.05	0.19	0.04	0.14	0.01	0.32	0.01
303	Levoglucosan	874.2640	161.05	0.18	0.02	0.24	0.04	0.14	0.06	0.31	0.05
304	Benzoic acid	879.1010	121.03	0.40	0.04	0.39	0.09	0.44	0.13	1.23	0.36
305	Dihydrouracil	891.2780	113.02	0.54	0.26	0.28	0.04	0.16	0.01	0.49	0.08

CK, TR, TS and TT stand for roots in the control, 25 mg kg^{-1} Cd, 50 mg kg^{-1} Cd, and 100 mg kg^{-1} Cd added group (n = 3), respectively (the same below).

Table S2: Differential metabolites in *S. miltiorrhiza* roots between the control group and the Cd stress groups

NO.	Metabolites	RT (s)	Mass	TR vs CK			TS vs CK			TT vs CK		
				p	FC	VIP	*p*	FC	VIP	*p*	FC	VIP
1	2,2-Dimethylsuccinic acid	14.8230	145.05	–	–	–	–	–	–	0.00	2.16	1.15
2	Ethyl glucuronide	18.3728	221.07	–	–	–	0.00	0.33	1.23	0.02	2.05	1.04
3	Glucose 6-phosphate	39.4271	259.02	0.01	0.72	1.16	0.01	0.74	1.13	0.03	1.24	1.00
4	L-Aspartic acid	40.5494	132.03	0.01	0.89	1.15	0.00	1.35	1.24	0.00	1.51	1.14
5	2',4',6'-Trihydroxyacetophenone	40.5496	267.07	0.00	1.41	1.18	0.02	1.18	1.12	0.00	1.35	1.12
6	γ-Aminobutyric acid	41.6726	102.06	0.00	0.63	1.23	–	–	–	0.00	1.30	1.14
7	Gluconic acid	41.6852	195.05	0.00	0.56	1.24	–	–	–	–	–	–
8	D-2,3-Dihydroxypropanoic acid	41.6877	105.02	–	–	–	–	–	–	0.02	1.44	1.04
9	β-Alanine	42.7915	88.04	0.00	1.30	1.20	0.00	1.66	1.25	0.00	1.21	1.10
10	Glycine	42.8068	74.02	–	–	–	–	–	–	0.00	2.39	1.14
11	L-Asparagine	42.8099	131.05	0.00	0.56	1.24	0.00	1.30	1.25	–	–	–
12	myo-Inositol	43.8189	179.06	0.00	1.22	1.22	0.00	1.49	1.26	0.00	1.45	1.15
13	D-Glutamine	43.9210	145.06	0.00	0.53	1.24	0.00	0.88	1.20	0.00	1.27	1.14
14	1,3,7-Trimethyluric acid	43.9228	209.07	0.00	0.55	1.24	0.00	0.77	1.23	0.00	0.95	1.11
15	Gluconolactone	43.9396	177.04	0.03	1.19	1.05	0.01	1.28	1.17	0.01	1.77	1.07
16	L-Histidine	43.9413	154.06	0.04	0.85	1.04	0.00	2.07	1.24	0.00	2.85	1.15
17	Glucobrassicin	45.0531	119.03	0.00	2.09	1.24	–	–	–	–	–	–
18	Pyrrolidonecarboxylic acid	46.1993	128.03	0.00	0.62	1.17	–	–	–	–	–	–
19	D-Glucurono-6,3-lactone	47.2963	175.02	0.01	1.35	1.11	0.00	1.27	1.20	0.00	2.39	1.14
20	L-Proline	47.3289	114.06	0.00	0.83	1.22	0.00	4.59	1.22	0.00	4.43	1.10
21	Oxoadipic acid	48.4517	159.03	–	–	–	–	–	–	0.02	2.08	1.02
22	1-Kestose	49.5215	503.16	0.00	0.76	1.22	0.00	0.72	1.25	0.01	1.13	1.07
23	Citraconic acid	49.5447	129.02	–	–	–	–	–	–	0.00	1.83	1.15

(*Continued*)

Table S2: *Continued*

NO.	Metabolites	RT (s)	Mass	TR vs CK			TS vs CK			TT vs CK		
				p	FC	VIP	*p*	FC	VIP	*p*	FC	VIP
24	Citramalic acid	49.5999	147.03	0.00	0.31	1.23	0.00	0.33	1.24	0.00	0.54	1.11
25	But-2-enoic acid	50.5531	85.03	–	–	–	0.00	2.59	1.23	0.01	3.27	1.08
26	2-Furoic acid	50.6292	111.01	–	–	–	0.00	0.72	1.23	0.00	1.63	1.09
27	5-Aminopentanoic acid	50.7066	116.07	0.00	1.87	1.23	0.00	3.01	1.26	0.00	1.92	1.15
28	L-Tyrosine	51.8316	180.07	0.00	2.79	1.23	0.00	4.73	1.25	–	–	–
29	Uridine	52.5865	243.06	–	–	–	0.00	0.42	1.22	0.00	0.22	1.14
30	Succinic acid	54.0272	117.02	0.00	0.55	1.24	0.00	0.55	1.26	–	–	–
31	Succinic anhydride	54.0943	99.01	0.00	0.74	1.23	0.00	0.51	1.24	–	–	–
32	Mannitol	54.0943	181.07	–	–	–	0.00	1.69	1.22	0.01	0.44	1.06
33	L-Serine	59.8012	104.03	0.01	1.42	1.14	0.00	1.83	1.24	0.02	3.45	1.04
34	Maltopentaose	71.3485	827.27	0.00	1.88	1.19	–	–	–	–	–	–
35	D-Tartaric acid	71.8595	149.01	–	–	–	–	–	–	0.01	1.76	1.04
36	Fumaric acid	72.2048	115.00	–	–	–	–	–	–	0.00	1.73	1.15
37	Stachyose	72.3970	665.22	0.00	1.84	1.20	0.03	0.61	1.08	0.00	0.03	1.13
38	L-Gulonolactone	73.4586	177.04	–	–	–	0.02	0.91	1.13	0.00	1.68	1.11
39	Raffinose	75.0982	503.16	0.00	1.56	1.17	–	–	–	–	–	–
40	Syringic acid	78.1883	197.05	0.00	1.78	1.20	0.00	3.17	1.23	–	–	–
41	Pyridoxal	79.0455	166.05	0.04	0.57	1.02				–	–	–
42	L-Norleucine	83.5828	130.09	0.00	1.44	1.21	0.00	1.86	1.24	0.00	1.28	1.10
43	Itaconic acid	84.0399	129.02	–	–	–	0.00	0.64	1.20	0.01	1.56	1.06
44	(2R)-6,8-Diglucopyranosyl-4',5,7-trihydroxyflavanone	85.0693	595.17	–	–	–	0.01	3.66	1.18	–	–	–
45	DL-Dopa	85.5300	196.06	0.00	2.57	1.23	0.00	5.21	1.26	–	–	–
46	Acetaminophen	85.7146	150.06	0.00	2.68	1.24	0.00	5.13	1.26	–	–	–
47	α-Ketoisovaleric acid	91.2160	115.04	0.00	1.16	1.22	0.00	0.35	1.19	–	–	–
48	Vanillin	99.4340	151.04	0.00	1.33	1.24	0.01	1.42	1.14	0.02	2.15	1.01
49	Trehalose	149.7160	341.11	–	–	–	0.00	0.47	1.22			
50	Erythrono-1,4-lactone	153.2710	117.02	–	–	–	0.05	1.28	1.03	0.00	2.84	1.13
51	Guanosine	187.4840	282.08	0.00	0.74	1.23	0.00	0.45	1.26	0.00	0.40	1.11
52	o-Tyrosine	190.6770	180.07	0.00	3.18	1.24	0.00	3.92	1.25	0.00	2.13	1.16
53	βmipron	190.6770	192.07	0.00	2.69	1.24	0.00	2.99	1.25	0.00	2.01	1.14
54	L-Phenylalanine	190.6785	164.07	0.00	2.97	1.24	0.00	3.21	1.26	0.00	2.31	1.16
55	Pantothenic acid	191.4645	218.10	0.00	1.17	1.18	0.00	1.40	1.24	0.00	0.29	1.13
56	N1-(2-Hydroxyethyl)flurazepam	192.8875	331.07	0.00	1.47	1.19	0.00	2.07	1.23	–	–	–
57	Imidazoleacetic acid	194.1700	125.04	0.00	1.30	1.19	0.00	1.53	1.25	0.00	2.45	1.15
58	L-Arginine	205.9810	173.10	0.00	0.85	1.19	0.00	3.27	1.26	0.00	1.87	1.11
59	Mycophenolic acid	218.0615	167.03	0.00	0.38	1.22	0.01	0.54	1.17	0.00	0.15	1.14
60	Terephthalic acid	222.2230	165.02	–	–	–	0.00	1.38	1.24	–	–	–
61	D-Malic acid	223.3700	133.01	–	–	–	0.03	1.24	1.18	0.00	1.61	1.15

(*Continued*)

Table S2: *Continued*

NO.	Metabolites	RT (s)	Mass	TR vs CK			TS vs CK			TT vs CK		
				p	FC	VIP	*p*	FC	VIP	*p*	FC	VIP
62	Phenobarbital	223.4740	231.08	–	–	–	0.00	4.08	1.25	0.01	1.90	1.08
63	5-Hydroxy-L-tryptophan	223.6080	219.08	0.00	1.50	1.21	0.00	4.36	1.25	0.00	2.03	1.13
64	Chorismate	225.2405	225.04	0.00	3.34	1.23	0.00	8.21	1.26	0.00	3.32	1.14
65	Estrone sulfate	229.4680	349.11	0.00	0.84	1.17	0.00	2.30	1.25	0.00	1.55	1.12
66	Phenylethyl primeveroside	229.9560	461.17	–	–	–	–	–	–	0.02	1.79	1.03
67	γ-Glutamylcysteine	231.3570	249.05	0.00	1.69	1.19	0.00	1.75	1.25	0.00	1.66	1.15
68	3,4-Dihydroxyhydrocinnamic acid	231.5800	181.05	0.00	1.45	1.23	0.00	1.42	1.25	0.00	1.76	1.16
69	3-(3,4-Dihydroxy-5-methoxy)-2-propenoic acid	232.6030	209.05	0.00	1.24	1.19	–	–	–	0.00	2.47	1.16
70	Dopamine	234.7840	299.08	0.00	0.63	1.22	0.01	1.50	1.15	0.02	1.23	1.04
71	Apiin	237.0730	563.14	0.00	1.84	1.22	0.00	2.77	1.21	0.00	3.06	1.14
72	(Ã Â±)-Tryptophan	240.3660	203.08	–	–	–	0.01	11.19	1.15	–	–	–
73	Trans-3-coumarate	241.2045	163.04	0.02	1.81	1.11	–	–	–	–	–	–
74	5,7-dihydroxy-2-(4-methoxyphenyl)-3,4-dihydro-2H-1-benzopyran-4-one	242.7500	285.08	0.00	0.34	1.24	0.00	0.29	1.26	0.00	0.20	1.16
75	p-Anisic acid	245.5660	151.04	0.02	1.35	1.10	0.01	1.42	1.18	0.00	1.77	1.14
76	3,4-Dihydroxy-trans-cinnamate	246.5080	179.03	0.00	1.10	1.21	0.00	1.59	1.26	0.00	2.10	1.16
77	3-Carboxy-4-methyl-5-propyl-2-furanpropionic acid	246.7470	239.09	0.00	0.77	1.17	0.01	0.75	1.16	–	–	–
78	3-(4-hydroxy-3-methoxyphenyl)prop-2-enoic acid	247.7055	193.05	0.03	1.49	1.05	0.00	1.97	1.20	–	–	–
79	3-Methoxyanthranilate	249.9430	166.05	0.01	1.18	1.17	–	–	–	0.00	0.83	1.13
80	Quercetin 3-O-(6''-acetyl-glucoside)	253.5360	505.10	0.00	0.39	1.22	–	–	–	0.00	0.17	1.15
81	Caffeic acid	260.4550	179.03	0.00	1.34	1.20	0.00	1.97	1.25	0.00	3.05	1.16
82	2-Methylbenzoic acid	260.4550	135.04	0.00	1.51	1.21	0.01	1.86	1.14	0.00	3.09	1.16
83	Rosmarinic acid	264.9790	359.08	0.01	1.20	1.16	0.00	3.46	1.25	0.00	6.00	1.16
84	Umbelliferone	264.9800	161.02	0.02	1.11	1.10	0.00	3.18	1.26	0.00	5.84	1.16
85	Aesculetin	265.0220	177.02	0.01	1.50	1.14	0.00	1.93	1.23	–	–	–
86	1,11-Undecanedicarboxylic acid	265.8720	243.16	0.00	0.60	1.21	0.01	1.22	1.14	0.00	0.30	1.11
87	Hydroxyphenyllactic acid	267.3150	181.05	0.00	1.50	1.23	0.00	1.94	1.26	0.00	2.49	1.16
88	(R)-Kawain	274.1690	229.09	0.00	0.30	1.23	–	–	–	–	–	–
89	1,2,3-Trihydroxybenzene	275.4195	125.02	–	–	–	0.00	0.73	1.22	0.00	1.42	1.15
90	Eupatilin	278.8890	343.08	0.00	0.42	1.24	0.00	1.18	1.26	0.00	1.43	1.13
91	5-Methoxysalicylic acid	284.8570	167.03	0.00	2.38	1.24	0.00	1.88	1.25	0.00	2.61	1.16
92	Theaflavin	284.9650	563.12	0.01	1.80	1.13	–	–	–	–	–	–
93	(R)-2-Benzylsuccinate	285.9225	207.07	0.03	0.78	1.08	0.01	0.79	1.15	0.00	0.57	1.14
94	Xanthoxylin	287.2465	195.07	–	–	–	–	–	–	0.00	3.59	1.16
95	Isoferulic acid	289.4125	193.05	0.00	0.70	1.17	0.03	0.88	1.08	0.01	0.73	1.08
96	Eudesmic acid	294.0770	211.06	0.00	2.89	1.24	0.00	2.83	1.25	0.00	8.18	1.16
97	Genipin	298.2050	225.08	0.00	3.36	1.23	0.02	1.95	1.12	0.00	2.54	1.15
98	Glycitein	299.0045	283.06	0.00	0.44	1.22	–	–	–	0.00	0.50	1.13

(*Continued*)

Table S2: *Continued*

NO.	Metabolites	RT (s)	Mass	TR vs CK			TS vs CK			TT vs CK		
				p	FC	VIP	*p*	FC	VIP	*p*	FC	VIP
99	Aspalathin	299.7360	451.13	0.01	0.52	1.15	0.00	3.00	1.23	0.00	4.93	1.15
100	Azelaic acid	304.3050	187.10	–	–	–	0.00	0.73	1.24	0.00	1.87	1.12
101	5,7-dihydroxy-2-(4-hydroxyphenyl)-6,8-dimethyl-3,4-dihydro-2H-1-benzopyran-4-one	306.6300	299.09	0.00	0.31	1.22	–	–	–	0.00	0.23	1.15
102	Byssochlamic acid	311.4040	331.12	0.01	0.44	1.16	–	–	–	–	–	–
103	Methyl vanillate	312.2720	181.05	–	–	–	–	–	–	0.01	1.26	1.07
104	γ-CEHC	314.9725	263.13	0.00	0.17	1.23	–	–	–	0.00	0.55	1.14
105	Geranylgeranyl-PP	315.0070	449.18	0.00	0.10	1.22	0.00	0.23	1.23	–	–	–
106	Glaucarubin	317.2550	495.22	–	–	–	0.00	1.79	1.25	0.01	1.68	1.09
107	Meconine	321.9640	193.05	0.00	1.72	1.17	0.04	1.31	1.03	0.00	1.86	1.13
108	Benazeprilat	325.3360	395.16	0.00	0.08	1.24	0.00	0.48	1.25	0.00	0.24	1.16
109	Corticosterone	330.0290	381.18	0.00	0.07	1.23	0.00	0.36	1.23	0.00	0.11	1.15
110	Gibberellin A3	331.2010	345.14	0.00	0.36	1.23	–	–	–	0.00	0.50	1.15
111	Cohumulone	331.2010	345.14	0.00	0.36	1.24	–	–	–	0.00	0.54	1.16
112	Aflatoxin B1	332.3690	311.06	0.00	0.65	1.22	–	–	–	0.00	0.61	1.11
113	trans-Piceid	335.4460	403.14	–	–	–	–	–	–	0.00	2.12	1.16
114	Ascochitine	340.3810	275.09	0.00	0.39	1.23	–	–	–	0.00	0.36	1.15
115	Glycitin	345.7380	445.11	–	–	–	–	–	–	0.00	2.10	1.14
116	Monoethyl phthalate	347.2630	193.05	0.00	0.84	1.18	0.00	1.29	1.20	0.00	1.49	1.16
117	5,7-dihydroxy-2-(4-hydroxy-3,5-dimethoxyphenyl)-4H-chromen-4-one	348.4230	329.07	–	–	–	0.02	1.49	1.12	–	–	–
118	Traumatic acid	351.8855	227.13	–	–	–	–	–	–	0.02	2.18	1.03
119	Myricetin 3-neohesperidoside	353.0460	625.14	–	–	–	–	–	–	0.00	2.08	1.15
120	Propionic acid	353.8485	73.03	–	–	–	–	–	–	0.00	2.22	1.15
121	Gibberellin A19	361.1740	361.17	0.00	0.28	1.23	0.00	1.32	1.21	0.00	0.49	1.14
122	(S,E)-Zearalenone	361.1810	317.14	0.00	0.40	1.20	–	–	–	–	–	–
123	Docosahexaenoic acid	373.8675	327.23	0.02	0.26	1.08	–	–	–	–	–	–
124	3-Hydroxycapric acid	374.5355	187.13	–	–	–	–	–	–	0.00	2.16	1.13
125	3-Hydroxyflavone	383.3590	237.06	0.00	0.47	1.23	0.01	1.20	1.14	0.00	0.54	1.15
126	Citric acid	387.8450	191.02	0.00	0.72	1.19	–	–	–	–	–	–
127	Methyl 2-hydroxybenzoate	392.5040	151.04	–	–	–	–	–	–	0.00	1.94	1.15
128	Indoleacetaldehyde	392.5070	158.06	0.00	1.54	1.23	0.00	3.43	1.26	0.00	1.60	1.15
129	bicyclo-PGE2	397.1400	333.21	0.00	0.31	1.23	0.01	0.70	1.17	0.00	0.60	1.12
130	Mono-benzyl phthalate	398.0830	255.07	0.00	0.20	1.22	0.00	1.36	1.19	0.00	2.01	1.14
131	Levocetirizine	401.7090	387.15	0.00	0.19	1.23	0.00	0.59	1.24	0.00	0.33	1.13
132	Heptanoic acid	402.8625	129.09	–	–	–	–	–	–	0.00	2.13	1.14
133	8-iso-15-keto-PGE2	407.4190	349.20	0.00	0.15	1.24	0.00	0.44	1.25	0.00	0.21	1.16
134	Leukotriene B4	413.8660	335.22	0.02	0.28	1.08	–	–	–	–	–	–
135	6β-Hydroxyasiatic acid	414.6740	503.34	0.00	0.17	1.24	0.00	0.61	1.26	0.00	0.36	1.16

(*Continued*)

Table S2: *Continued*

NO.	Metabolites	RT (s)	Mass	TR vs CK			TS vs CK			TT vs CK		
				p	FC	VIP	*p*	FC	VIP	*p*	FC	VIP
136	Daidzin	415.7995	461.11	–	–	–	–	–	–	0.00	2.43	1.14
137	13-OxoODE	419.3375	319.12	0.00	0.12	1.23	0.00	0.64	1.25	0.00	0.30	1.16
138	Isokobusone	421.6130	347.19	0.00	1.26	1.20	–	–	–	0.00	1.39	1.14
139	[6]-Dehydrogingerdione	428.8840	289.15	0.01	0.49	1.16	0.00	2.20	1.20	–	–	–
140	Formononetin	430.0605	267.07	0.00	0.33	1.24	0.00	0.65	1.22	0.00	0.32	1.16
141	Caprylic acid	430.6780	143.11	–	–	–	–	–	–	0.00	1.98	1.14
142	[8]-Dehydrogingerdione	433.4030	317.18	0.00	0.40	1.24	–	–	–	–	–	–
143	Butylparaben	439.9290	193.09	0.02	1.42	1.11	–	–	–	0.00	2.19	1.15
144	Gingerol	440.5970	293.18	0.00	1.32	1.24	0.02	0.92	1.12	0.00	1.50	1.16
145	Inosine	441.0850	221.15	–	–	–	0.02	1.22	1.11	0.00	1.59	1.13
146	Secoisolariciresinol	443.0875	361.17	0.00	0.17	1.21	–	–	–	–	–	–
147	Gibberellin A44	443.7450	345.17	0.00	0.28	1.22	0.04	0.75	1.05	0.00	0.49	1.13
148	4-hydroxy-3-[1-(4-hydroxyphenyl)-3-oxobutyl]-2H-chromen-2-one	445.5930	323.09	0.00	0.60	1.23	0.00	0.66	1.24	0.00	0.33	1.16
149	(-)-Matairesinol	445.9630	357.13	0.00	0.53	1.17	0.01	0.54	1.18	0.02	0.67	1.04
150	4-[(E)-2-(3,5-dimethoxyphenyl)ethenyl]phenol	449.9785	255.10	0.00	0.19	1.23	–	–	–	0.00	0.24	1.15
151	Methyl jasmonate	450.7100	223.13	–	–	–	0.00	2.04	1.26	0.01	1.98	1.06
152	2-Hydroxyestradiol	454.0490	287.17	0.00	0.20	1.24	0.00	1.49	1.24	0.00	1.32	1.13
153	Medicagenic acid	458.6755	501.32	0.01	0.18	1.15	0.01	1.77	1.16	–	–	–
154	(R)-3-Hydroxy-tetradecanoic acid	458.6940	243.20	0.03	1.21	1.05	0.04	0.87	1.05	–	–	–
155	Prostaglandin D3	463.2940	349.20	0.00	0.25	1.23	–	–	–	0.00	0.40	1.15
156	Methylgingerol	466.2470	307.19	0.00	0.13	1.24	–	–	–	0.00	0.37	1.16
157	8-iso-PGA1	469.0025	335.22	0.00	0.23	1.18	–	–	–	–	–	–
158	Leukotriene B5	472.6090	333.21	0.00	0.23	1.24	0.00	0.66	1.23	0.00	0.21	1.16
159	Resolvin D2	473.9210	375.22	–	–	–	0.00	5.74	1.23	–	–	–
160	15-Deoxy-d-12,14-PGJ2	475.0145	315.20	0.00	0.22	1.24	0.00	0.65	1.25	0.00	0.21	1.16
161	Pelargonic acid	475.9910	157.12	–	–	–	–	–	–	0.00	1.38	1.16
162	Tetrahydrocurcumin	476.1150	371.15	0.05	0.45	1.01	0.00	2.86	1.23	–	–	–
163	α-Linolenic acid	480.0740	277.22	0.00	0.07	1.24	0.00	0.36	1.25	–	–	–
164	4',5,7-Trihydroxy-6-prenylflavanone	482.0050	339.12	0.00	0.50	1.22	0.00	0.73	1.25	0.00	0.35	1.16
165	Cortisone	483.6220	359.19	0.01	0.30	1.12	0.05	0.46	1.03	–	–	–
166	Dihydrojasmonic acid	485.8090	211.13	0.02	1.09	1.09	–	–	–	0.00	2.59	1.16
167	Gibberellin A53	489.2330	347.19	0.02	0.19	1.11	–	–	–	–	–	–
168	8,15-DiHETE	490.5355	335.22	–	–	–	0.02	1.81	1.12	–	–	–
169	Ethyl hexadecanoate	495.8925	283.26	–	–	–	0.01	0.10	1.15	0.01	3.27	1.09
170	20-Hydroxyeicosatetraenoic acid	496.1255	319.23	0.00	0.45	1.24	0.00	1.85	1.26	0.00	0.63	1.15
171	6β-Hydroxytestosterone	500.4950	303.20	0.00	0.22	1.24	–	–	–	0.00	0.27	1.16
172	Linoleic acid	502.5945	279.23	0.00	0.07	1.24	0.00	0.36	1.26	0.02	0.93	1.02
173	4-Dodecylbenzenesulfonic Acid	504.9300	325.18	–	–	–	–	–	–	0.02	1.52	1.03

(*Continued*)

Table S2: *Continued*

NO.	Metabolites	RT (s)	Mass	TR vs CK			TS vs CK			TT vs CK		
				p	FC	VIP	*p*	FC	VIP	*p*	FC	VIP
174	Estriol	506.4725	287.17	0.00	0.20		0.00	0.59	1.25	0.02	0.86	1.02
175	Resolvin D1	507.6850	375.22	0.00	0.04	1.24	0.00	0.35	1.26	0.00	0.35	1.16
176	Geranylacetone	509.2375	329.29	0.03	0.52	1.06	0.02	0.44	1.09	–	–	–
177	Thyrotropin releasing hormone	510.6950	361.17	0.03	0.36	1.07	–	–	–	–	–	–
178	LysoPA(16:0/0:0)	512.1710	409.24	0.01	0.30	1.13	–	–	–	0.00	0.27	1.12
179	Esculentic acid (Diplazium)	514.0910	487.34	0.00	0.18	1.24	0.00	0.50	1.26	0.00	0.73	1.15
180	11-Dehydrocorticosterone	518.9910	343.19	0.00	0.19	1.23	0.04	0.81	1.04	0.00	0.35	1.14
181	Estradiol	520.1230	271.17	0.00	0.14	1.24	0.00	0.61	1.26	0.01	1.10	1.08
182	5-HEPE	523.3710	317.21	0.00	0.24	1.24	0.00	0.57	1.25	0.00	0.28	1.16
183	2',5,6-Trimethoxyflavone	524.4140	311.09	0.00	0.47	1.20	–	–	–	–	–	–
184	11(R)-HETE	524.6510	319.23	0.00	0.85	1.18	0.00	1.51	1.25	0.00	0.72	1.12
185	Cortisol	526.6860	361.20	0.00	0.15	1.23	0.04	0.62	1.04	0.00	0.43	1.15
186	[10]-Dehydrogingerdione	529.0950	345.21	0.00	0.14	1.24	0.00	0.52	1.22	0.00	0.32	1.16
187	Ethyl dodecanoate	532.6140	227.20	–	–	–	–	–	–	0.00	2.71	1.16
188	(10E,12Z)-9-HODE	534.7250	295.23	0.00	0.12	1.24	0.00	0.23	1.26	0.00	0.24	1.16
189	Undecanoic acid	540.5345	185.15	–	–	–	0.02	1.10	1.12	0.00	2.13	1.15
190	Carnosol	545.7535	329.18	0.00	0.51	1.24	0.04	1.51	1.04	–	–	–
191	1,7-bis(4-hydroxyphenyl)-5-methoxyheptan-3-one	546.3315	327.16	0.00	0.60	1.22	0.00	0.45	1.23	0.00	0.50	1.15
192	(2E)-3-(2-hydroxyphenyl)-1-(4-methoxyphenyl) prop-2-en-1-one	547.4480	253.09	0.00	0.36	1.20	0.01	0.56	1.17	–	–	–
193	Dehydroepiandrosterone	547.4790	287.20	0.00	0.25	1.23	0.00	0.80	1.21	0.00	0.31	1.15
194	18R-HEPE	550.8900	317.20	0.00	0.22	1.23	0.00	0.79	1.24	0.00	0.37	1.10
195	L-Erythrulose	552.0260	293.21	–	–	–	–	–	–	0.00	2.44	1.14
196	Tridecanoic acid	552.5855	213.19	–	–	–	–	–	–	0.02	2.39	1.03
197	9-cis-Retinoic acid	553.1475	299.20	0.00	0.38	1.24	0.01	0.91	1.16	0.00	0.67	1.16
198	L-Malic acid	558.8125	133.01	0.00	0.76	1.17	0.00	0.51	1.24	–	–	–
199	2-Methoxyestrone	558.8540	299.17	0.00	0.41	1.23	–	–	–	–	–	–
200	16-Hydroxy hexadecanoic acid	558.8590	271.23	–	–	–	–	–	–	0.01	2.11	1.08
201	Estrone	559.9470	269.15	0.00	0.27	1.23	0.00	0.44	1.24	0.01	0.67	1.08
202	Palmitic acid	562.2520	255.23	0.01	1.14	1.15	0.00	0.66	1.20	0.00	2.58	1.15
203	all-trans-Retinoic acid	567.8640	299.20	0.00	0.45	1.22	–	–	–	–	–	–
204	Dodecanoic acid	570.1730	199.17	0.01	1.18	1.15	0.00	1.23	1.19	0.00	2.22	1.15
205	Steviobioside	575.3325	641.31	0.00	0.23	1.20	–	–	–	0.00	0.41	1.12
206	γ-Glutamylmethionine	575.9740	277.09	0.00	0.57	1.19	–	–	–	0.00	0.48	1.14
207	Eicosapentaenoic acid	579.3335	301.22	0.00	0.47	1.22	–	–	–	–	–	–
208	Isopalmitic acid	579.8880	255.23	–	–	–	0.00	0.69	1.21	0.00	2.51	1.15
209	12-HEPE	581.4510	317.21	0.00	0.42	1.24	–	–	–	0.02	0.88	1.04
210	2,3-Dihydroxybutanedioic acid	581.5135	149.01	–	–	–	–	–	–	0.00	2.00	1.16
211	Myristoleic acid	589.8555	225.19	–	–	–	0.01	1.34	1.16	0.01	1.62	1.05

(*Continued*)

Table S2: *Continued*

NO.	Metabolites	RT (s)	Mass	TR vs CK			TS vs CK			TT vs CK		
				p	FC	VIP	*p*	FC	VIP	*p*	FC	VIP
212	Carnosic acid	590.6010	331.19	0.00	0.20	1.24	0.02	1.14	1.13	0.00	0.36	1.16
213	Maslinic acid	595.1580	471.35	0.00	0.25	1.24	0.00	0.34	1.26	–	–	–
214	Arachidonic acid	597.4330	303.23	0.00	0.48	1.21	–	–	–	0.01	0.66	1.09
215	2-Ketobutyric acid	599.6950	101.02	–	–	–	0.04	0.42	1.04	–	–	–
216	21-Hydroxypregnenolone	605.3220	331.23	0.00	0.12	1.24	0.00	0.64	1.24	0.00	0.11	1.16
217	Dehydroabietic acid	605.6125	299.20	0.01	0.22	1.13	–	–	–	0.02	0.37	1.01
218	Cholesterol sulfate	609.4680	465.31	0.01	0.33	1.16	0.01	0.34	1.15	–	–	–
219	15-KETE	609.8380	317.21	0.00	0.05	1.24	0.00	0.34	1.26	0.00	0.08	1.16
220	Glycyrrhetinic acid	620.0490	469.33	0.00	0.22	1.24	0.00	0.46	1.26	0.00	0.57	1.16
221	Suberic acid	623.1150	173.08	0.01	1.42	1.15	–	–	–	0.00	2.88	1.16
222	Myristic acid	624.5770	227.20	0.04	0.89	1.05	–	–	–	0.00	1.72	1.13
223	16(17)-EpDPE	639.2690	343.23	–	–	–	–	–	–	0.00	0.14	1.14
224	Epiandrosterone	640.4580	289.22	0.00	0.39	1.23	0.01	0.78	1.16	0.00	0.28	1.15
225	Pentadecanoic acid	641.5060	241.22	–	–	–	–	–	–	0.00	1.82	1.14
226	Bovinic acid	644.9405	279.23	0.00	0.15	1.24	0.00	0.27	1.26	0.00	0.46	1.16
227	Ursolic acid	644.9410	455.35	0.00	0.28	1.23	0.00	0.50	1.24	–	–	–
228	3-Methyladipic acid	659.0490	159.07	–	–	–	–	–	–	0.01	1.90	1.05
229	Ethyl tetradecanoate	669.7930	255.23	0.00	0.49	1.23	0.00	0.66	1.24	–	–	–
230	Oleic acid	676.5640	281.25	0.00	0.19	1.23	0.00	0.39	1.25	0.00	0.48	1.15
231	Salicyluric acid	678.8300	194.05	–	–	–	0.02	1.91	1.12	0.01	3.03	1.06
232	Porphobilinogen	681.6780	225.09	–	–	–	–	–	–	0.01	2.19	1.06
233	15-Methylpalmitate	693.4880	269.25	0.00	0.41	1.23	–	–	–	–	–	–
234	Allocystathionine	697.9965	221.06	–	–	–	–	–	–	0.00	2.51	1.12
235	2,6-Pyridinedicarboxylic acid	743.1530	166.02	–	–	–	–	–	–	0.01	4.09	1.04
236	2-Furoylglycine	746.4990	168.03	–	–	–	–	–	–	0.02	2.52	1.02
237	4-Pyridoxic acid	761.1675	182.05	–	–	–	0.02	1.71	1.11	0.01	3.41	1.09
238	D-Xylulose	776.8875	149.05	–	–	–	0.04	3.55	1.05	0.01	2.16	1.05
239	5-Methylcytosine	779.2965	124.05	–	–	–	–	–	–	0.00	2.59	1.15
240	Acetylisoniazid	780.3070	178.06	–	–	–	–	–	–	0.00	2.21	1.13
241	Pyruvic acid	780.4235	87.01	–	–	–	0.01	0.79	1.15	0.00	2.20	1.14
242	3-Furoic acid	784.8590	111.01	–	–	–	0.00	0.75	1.19	0.00	1.94	1.12
243	Gallic acid	784.8590	169.01	–	–	–	0.00	0.56	1.25	0.00	2.63	1.14
244	Phenylglyoxylic acid	786.1190	149.02	–	–	–	–	–	–	0.01	2.22	1.07
245	(R)-lipoic acid	786.7375	223.02	–	–	–	–	–	–	0.00	2.71	1.14
246	Stearic acid	793.6710	283.26	–	–	–	0.00	0.23	1.23	0.00	2.95	1.14
247	Glyceraldehyde	804.2610	89.02	–	–	–	0.00	2.46	1.24	–	–	–
248	Quinic acid	809.1910	191.06	–	–	–	–	–	–	0.00	2.25	1.15
249	6-Hydroxynicotinic acid	820.5530	138.02	–	–	–	0.01	0.61	1.18	–	–	–
250	3-Methyl-2-oxovaleric acid	840.6390	129.06	0.02	1.28	1.10	0.02	0.80	1.10	0.00	2.54	1.13

(*Continued*)

Table S2: *Continued*

NO.	Metabolites	RT (s)	Mass	TR vs CK			TS vs CK			TT vs CK		
				p	**FC**	**VIP**	***p***	**FC**	**VIP**	***p***	**FC**	**VIP**
251	Phenylacetic acid	842.1030	135.04	–	–	–	–	–	–	0.02	2.47	1.03
252	Glutaric acid	843.0880	131.03	–	–	–	–	–	–	0.01	1.80	1.09
253	Pyrrole-2-carboxylic acid	856.8650	110.02	–	–	–	–	–	–	0.01	1.73	1.09

Differential metabolites in roots between control group and each Cd stress group are selected based on $p < 0.05$ from *t*-test, and VIP >1 from PLS-DA model; – stands for the data of the metabolites which were not the differential metabolites in roots; FC (fold change) stands for an estimate of the ratio of the relative content of metabolites in roots of the Cd stress group to that of control group.

Table S3: Results of pathway analysis involving all identified metabolites in *S. miltiorrhiza* roots with different levels of soil Cd stress

Pathway	Total Cmpd	Hits	Holm adjust	FDR	Impact
Isoquinoline alkaloid biosynthesis	6	3	1.00	0.80	1.00
Betalain biosynthesis	3	2	1.00	0.89	1.00
Linoleic acid metabolism	4	1	1.00	1.00	1.00
Alanine, aspartate and glutamate metabolism	22	8	0.80	0.40	0.65
C5-Branched dibasic acid metabolism	6	3	1.00	0.80	0.50
Phenylalanine metabolism	11	2	1.00	1.00	0.47
Cutin, suberine and wax biosynthesis	18	3	1.00	1.00	0.44
Glycine, serine and threonine metabolism	33	6	1.00	1.00	0.42
Valine, leucine and isoleucine biosynthesis	22	6	1.00	0.89	0.37
Butanoate metabolism	17	5	1.00	0.89	0.36
β-Alanine metabolism	18	5	1.00	0.89	0.33
Pyruvate metabolism	22	5	1.00	1.00	0.32
Glyoxylate and dicarboxylate metabolism	29	7	1.00	0.89	0.28
Pantothenate and CoA biosynthesis	23	6	1.00	0.89	0.26
Arginine and proline metabolism	34	4	1.00	1.00	0.25
Starch and sucrose metabolism	22	3	1.00	1.00	0.23
Tyrosine metabolism	16	6	1.00	0.60	0.22
Pentose and glucuronate interconversions	16	1	1.00	1.00	0.22
Citrate cycle (TCA cycle)	20	5	1.00	1.00	0.22
Vitamin B6 metabolism	11	1	1.00	1.00	0.21
Glutathione metabolism	26	4	1.00	1.00	0.19
Arginine biosynthesis	18	4	1.00	1.00	0.17
Phenylalanine, tyrosine and tryptophan biosynthesis	22	3	1.00	1.00	0.17
Galactose metabolism	27	4	1.00	1.00	0.15
Glycolysis/Gluconeogenesis	26	2	1.00	1.00	0.12
Pyrimidine metabolism	38	4	1.00	1.00	0.12
Aminoacyl-tRNA biosynthesis	46	10	1.00	0.89	0.11
α-Linolenic acid metabolism	28	2	1.00	1.00	0.11
Inositol phosphate metabolism	28	2	1.00	1.00	0.10

All pathways shown in the table are potential target metabolic pathways with pathway impacts of above 0.1; Total Cmpd, total number of compounds in the pathway; Hits, the number of actually matched compound in the pathway; Holm adjust, p value adjusted by Holm-Bonferroni method; FDR, p value adjusted using False Discovery Rate; Impact, pathway impact value.
